# Oligonucleotide aptamers for pathogen detection and infectious disease control

**DOI:** 10.7150/thno.61804

**Published:** 2021-08-27

**Authors:** Quanyuan Wan, Xiaohui Liu, Youli Zu

**Affiliations:** Department of Pathology and Genomic Medicine, Houston Methodist Hospital, Houston, TX 77030, USA

**Keywords:** aptamers, aptasensors, pathogen detection, disease control, immune system

## Abstract

During an epidemic or pandemic, the primary task is to rapidly develop precise diagnostic approaches and effective therapeutics. Oligonucleotide aptamer-based pathogen detection assays and control therapeutics are promising, as aptamers that specifically recognize and block pathogens can be quickly developed and produced through simple chemical synthesis. This work reviews common aptamer-based diagnostic techniques for communicable diseases and summarizes currently available aptamers that target various pathogens, including the SARS-CoV-2 virus. Moreover, this review discusses how oligonucleotide aptamers might be leveraged to control pathogen propagation and improve host immune system responses. This review offers a comprehensive data source to the further develop aptamer-based diagnostics and therapeutics specific for infectious diseases.

## Introduction

Communicable diseases are illnesses caused by pathogens, including viruses, bacteria, fungi, or parasitic protozoa that are disseminated among populations. Pathogens are usually microorganisms that are difficult to detect, with unhygienic living conditions facilitating pathogen infection. Transmission can occur through contact with contaminated surfaces or fluid, insect bites, food, water, and the air. Many communicable diseases share similar signs and symptoms. Diagnosis via laboratory tests, imaging scans, or biopsies is the first step in controlling communicable diseases. Laboratory tests, including microbiological methods, immunological methods, and PCR are widely available, broadly used, and reliable but do have disadvantages 1. Alternative diagnostic methods include aptamer-based diagnosis technologies, which are increasingly focused on the molecular diagnosis of diseases, because they are more cost effective, sensitive, specific, and convenient 2-4.

An oligonucleotide aptamer is a small single-stranded DNA (ssDNA) or RNA (ssRNA) that can self-fold into a specific 3D-spatial conformation 5. With these specific conformations, aptamers can bind to their targets exclusively through hydrogen bonding, electrostatic interactions, hydrophobic effects, π-π stacking, or van der Waals forces 6, 7. As such, aptamers are also termed 'chemical antibodies' and offer unique advantages compared to antibodies 8. Briefly, oligonucleotide aptamers offer batch consistency, stability at ambient temperatures, decreased immunogenicity, and opportunities for chemical modification to augment delivery characteristics. In the context of communicable disease diagnosis and treatment, aptamers can be rapidly developed against microbial toxins and nonimmunogenic components. As numerous oligonucleotide aptamers can be developed against diverse pathogens, aptamers are promising sensors and therapeutic agents for disease diagnosis and therapy, particularly for emerging communicable diseases.

This review describes the basic principle underlying commonly used oligonucleotide aptamer-based detection technologies, summarizes the detailed information of oligonucleotide aptamers targeting various pathogens, and introduces aptamer-based therapeutics for infectious diseases. Further we describe which aptamers have been developed against which kind of pathogens, how these aptamers were developed, how to design aptamer-based diagnostic methods, and the qualities that allow particular aptamers to be used for infectious disease diagnosis and control. This review aims to encourage and accelerate the development of aptamer-based diagnostics and therapeutics for infectious diseases.

## Potential utility of aptamer technology for pathogen detection

### Aptamer-based detection technologies

In this section, ultimate principles of aptamer-based detection technologies frequently used in published works were introduced, aiming at depicting brief mechanisms of various pathogen detection methods the reference of related researchers when developing a new detection method or improve a reported detection method.

#### Enzyme-linked oligonucleotide assay (ELONA)

**Figure 1:**
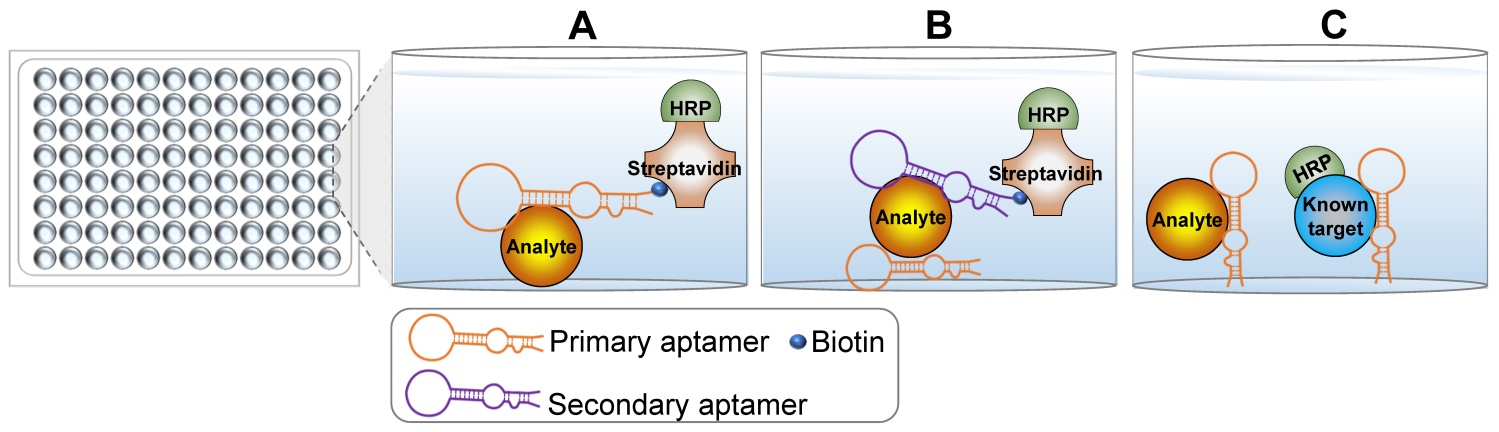
Schematic representation of the principle underlying ELONA. (A) Direct ELONA. Samples are coated on the bottom and detected using the biotin-labeled analyte-targeting aptamer and streptavidin-HRP. A stronger signal indicates more analytes. (B) Sandwich ELONA. An analyte-targeting aptamer is immobilized on the bottom and samples are added to the wells. Analytes are captured by the immobilized aptamers and detected using another analyte-targeting aptamer and streptavidin-HRP. A stronger signal indicates more analytes. (C) Competitive ELONA. Analyte-targeting aptamers are immobilized on the well plate bottom. Samples and a known HRP-conjugated standard are then added into the wells synchronously to allow the analytes to compete with the standard substance. The weaker signal indicates more analytes.

With the widespread development and commercialization of enzyme-linked immunosorbent assay (ELISA) technology, a mimic technology termed enzyme-linked oligonucleotide assay (ELONA) was developed to detect targets of interest 9, 10. The key difference between ELONA and ELISA is that ELONA uses aptamers as the main molecular recognition element instead of antibodies. Theoretically, any ELISA-based detection assay could be carried out using ELONA with the appropriate aptamers. The use of aptamers over antibodies in ELONA confers several advantages. For example, oligonucleotide aptamers are easily labeled with biotin or small molecule dyes that do not significantly impact the specificity and binding affinity for target molecules. As such, ELONA can be simply designed using a single labeled aptamer (Figure 1A). This configuration is less expensive than direct ELISA and simpler than indirect ELISA. Sandwich ELONA can be used to lessen the background noise originating from target immobilization in direct ELONA (Figure 1B). For the sandwich ELONA, two different aptamers that bind to unique sites on the same target are generated. One aptamer without a biotin label (primary/capture aptamer) is immobilized on a solid surface for target capture, while another biotinylated aptamer (secondary aptamer) is used for target detection. Notably, another advantage of ELONA is that both aptamers can be easily selected from different aptamer lineages using the systematic evolution of ligands by exponential enrichment (SELEX) procedure. Sandwich ELONA offers a favorable alternative for analytes lacking two recognition antibodies. Because the theoretical principle in target detection is similar, antibodies and aptamers can be used in conjunction with each other. The primary or secondary aptamer can be replaced by an antibody to form an aptamer-antibody-based enzyme-linked detection platform. Besides, competitive ELONA that operates under the same principles as competitive ELISA is developed (Figure 1C).

A key and challenging step for both sandwich and competitive ELONA is the immobilization of the primary aptamer on the surface of solid support 10. Primary aptamers should be sufficiently immobilized with reliable binding affinity and specificity for their targets. Several immobilization methods are described in previous books and review articles 11-13. Briefly, aptamers can be immobilized using physical and chemical reactions, including physical adsorption, covalent binding, self-assembly, avidin-biotin immobilization, and hybridization. An appropriate immobilization strategy renders ELONA an ideal assay for detecting targets of interest where antibodies have failed.

#### Aptamer-based lateral flow assay (ALFA)

**Figure 2:**
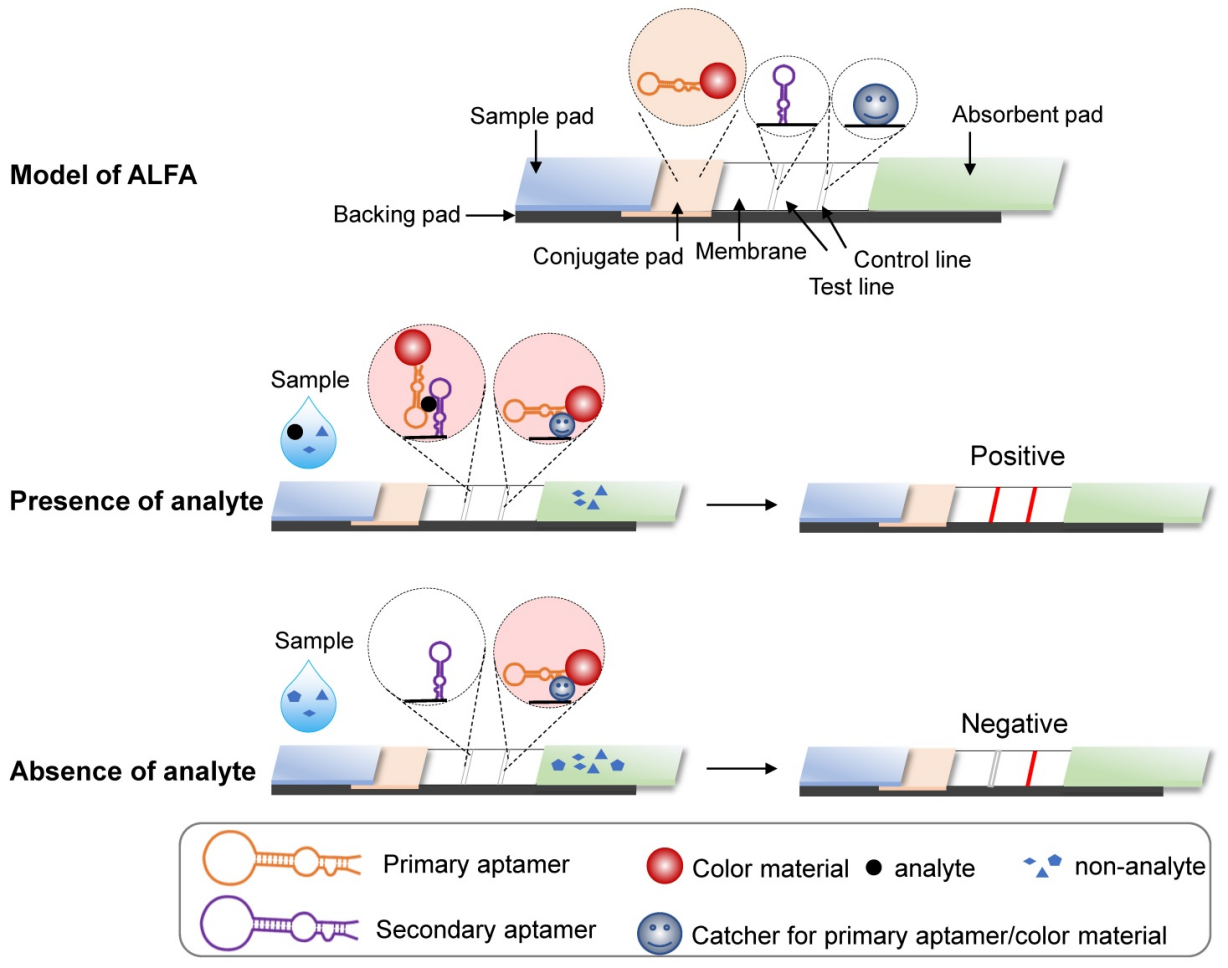
Schematic representation of ALFA. The basic principle is the same as in an antibody-based lateral flow assay. If the analyte of interest is present in the sample, the colored substance remains at the test and control lines. In contrast, if the analyte is absent, the colored substance only remains at the control line.

Aptamers can also be used for the lateral flow assay (LFA), a paper-based platform designed to detect targets in liquid samples 14. As in ELISA, affinity interactions underlie target capture at a visible line by an immobilized sensor. An LFA system is composed of five parts 14-17: a pad for sample loading; a conjugate pad containing the conjugate of target-specific recognition molecules with colored or fluorescent particles (CPs); a membrane immobilizing the sensor that recognizes targets at the test line and molecules that capture conjugate from the conjugate pad at the control line; an absorbent pad for wicking the sample liquid flow through the conjugate pad and the membrane; and a supportive backing pad for the system (Figure 2). Generally, LFAs can be divided into two formats: direct assay and competitive assay 15, 17. For direct assays, target-containing samples infiltrate the conjugate pad where targets bind recognition molecules conjugated with CPs. CPs are detained in the test line, as bound targets contain an immobilized sensor. The remaining CPs are captured and retained by the immobilized CPs binding molecules. As a result, both the test and control lines will be colored. However, if the sample lacks the target, only the control line becomes colored. For competitive format assays, the principle is the same as direct assays, but the result interpretation is reversed, i.e., a two-colored line result indicates negativity and a control-only line indicates positivity.

Typically, antibodies comprise both the specific recognition molecules in the conjugate pad and the target-binding biosensors and capture molecules on the membrane. Because nucleotide acid aptamers can recognize targets of interest specifically, antibodies for LFA can be replaced with aptamers. Aptamer-based LFAs (ALFA) offers a more flexible design and the ability to detect a wider range of targets. For example, sandwich ALFA should be considered for small molecules with few antigenic determinants, as these cannot be recognized by two different antibodies 18. Further, in ALFA, both capture molecules and the membrane-immobilized sensor at the test line can be antibodies, aptamers, complementary ssDNA probes, split aptamers, or conjugates, such as biotin or streptavidin 16, 17, 19. More promising and referential designs of ALFA were systematically introduced in the special topic review for nucleic acid aptamer-based lateral flow assays 20.

#### Aptasensor

**Figure 3:**
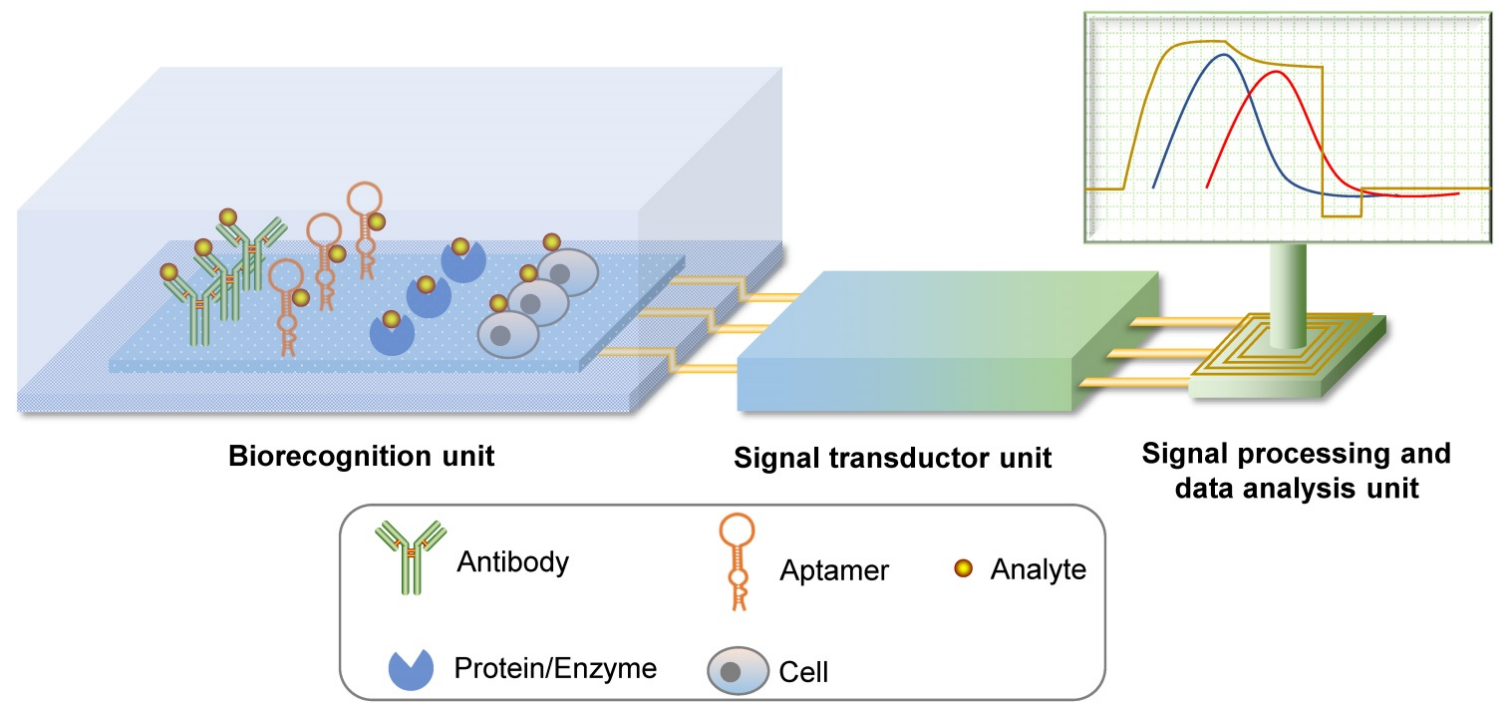
Schematic representation of biosensors. A biosensor system is comprised of a biorecognition unit, a signal transductor unit, and a signal processing and data analysis unit. Analytes can be recognized by their specific antibodies, aptamers, proteins or enzymes, and even cells. If the core recognition element is an aptamer, the biosensor system can be called aptasensor.

Biosensors are detection systems for biological analytes that contain a biorecognition sensing element, a signal transducer, and a signal processing unit (Figure 3). A specific biorecognition sensing element is the basis of a biosensor. This element can be immobilized antibodies, nucleic acid or protein aptamers, proteins, enzymes, or even cells 21, 22. By combining physical and chemical principles with computer techniques, biochemical reactions are transduced into digital signals that can be observed and analyzed directly. Biosensors that use aptamers as recognition elements are called aptasensors. Aptasensors are further categorized as optical, electrochemical, or mass-sensitive signal transducer types 21.

Optical aptasensors are generally divided into labeled and unlabeled aptamer models according to the aptamer modification. In a labeled aptamer model, aptamers are labeled with optically active molecules, such as fluorescent dyes, or colorimetric or luminescent materials. Based on the allosteric character of the aptamer after target binding, labeled optical molecules are either activated or quenched 23, 24 and aggregated or dispersed 25, 26, which changes the optical properties of the labeled molecules. In an unlabeled aptamer model, biorecognition induces a surface property change that is detected by an optical system 22. This model also makes use of physical findings, including surface plasmon resonance (SPR), evanescent wave fluorescence, and optical waveguide interferometry.

Electrochemical aptasensor principles have been reviewed previously 27. Briefly, a signal transducer unit transduces micro-current or micro-voltage changes into an observable signal. Biorecognition induces a conformational change in the aptamer or brings covalently linked enzymes onto the surface of the electrode. With redox molecules, such changes trigger a charge flux, which is then detected and quantified. The most commonly used electrochemical sensors are voltammetric (amperometric) and impedimetric sensors. To improve the sensitivity of electrochemical aptasensors, some strategies can be applied to amplify the singal which was thoroughly discussed in the previous review 28.

Mass-sensitive aptasensors are primarily known as quartz crystal microbalance (QCM) aptasensors that operate based on the piezoelectric effect. QCM detects mass changes on the crystal by measuring the change in frequency of a quartz crystal resonator 21. Aptamers are immobilized at one side of the crystal and the specific binding event is detected upon sensing the mass change. QCM aptasensors that detect various pathogens have also been developed 29-31.

#### Aptamer-based fluorophotometry

**Figure 4:**
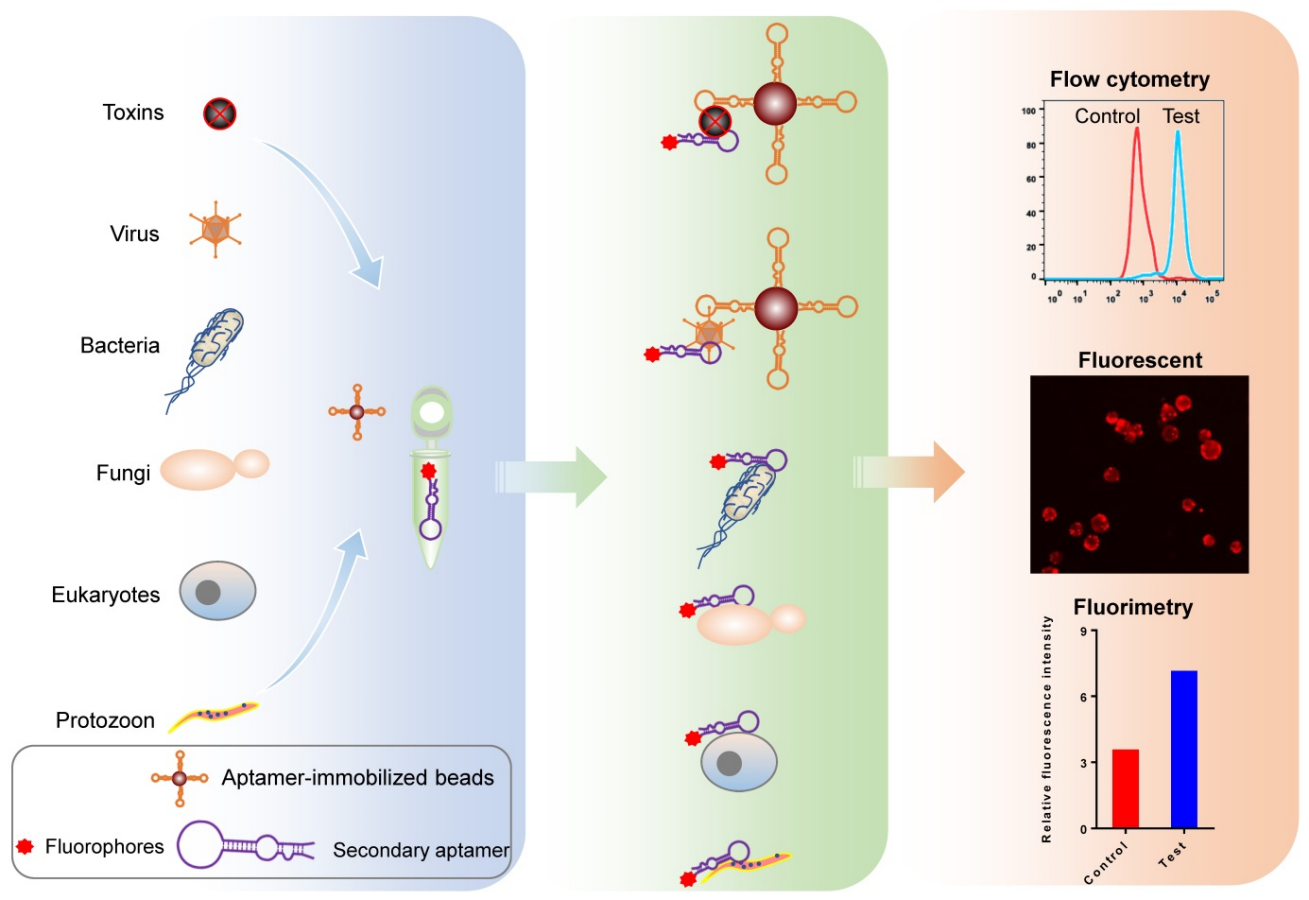
Schematic representation of pathogen detection using fluorophore-labeled aptamer probes. Fluorophore-conjugated or -labeled aptamers can be used as probes to detect pathogens and microbial toxins using various methods, including flow cytometry, fluorescent microscopy, and fluorimetry.

Fluorophore-labeled aptamers, aptamer-conjugated quantum dots (QD), or upconversion nanoparticles can be used as probes to detect targets of interest using a flow cytometer, fluorescent microscopy, or a fluorometer. A classic protocol for cancer cell detection involves labeling cancer cells using specific fluorophore-labeled aptamers obtained using cell-SELEX 32-34. Other small targets, such as microorganisms, toxins, and other biomolecules can also be Fluorophore-labeled using aptamers 35, 36. Figure 4 illustrates the use of aptamers to detect biomolecules and microorganisms (< 1 μm), such as viruses, chlamydia, and mycoplasma, which cannot be isolated using ordinary centrifugation techniques, aptamer-immobilized magnetic beads, or agarose beads. The intensity of the resulting fluorescent signal reflects the quantity of target present. Though aptamer-based fluorescent probe assays are low-cost, simple, rapid, and intuitive, they do have disadvantages, including: (1) aptamer immobilization; (2) dependence on the precision of experimental apparatuses; (3) a requirement for logical control setting; and (4) low sensitivity 37.

For structure-switching aptamers, some improvements have been developed based on fluorescence resonance energy transfer (FRET). Shen et al. split a DNA aptamer into two parts for target detection 38. One part was labeled with biotin and the other with a fluorophore. Upon target binding, the combined parts are captured by streptavidin-labeled beads, generating fluorescence. If a sample lacks the target, the streptavidin-labeled beads capture the biotin-labeled part only and beads remain non-fluorescent. Chinnappan et al. used graphene oxide (GO), an energy acceptor, to quench the fluoresce of the labeled aptamer 39. Once the aptamer binds its target, a structural change in the aptamer results in the removal of aptamer-conjugated fluorophores from GO, allowing fluorescence recovery. Other quenchers, such as TAMRA dye 23, cytochrome C protein 40, and gold nanoparticles (GNPs) 41 can be considered depending on the fluorescent materials used.

#### Amplification assay based on aptamer-mediated pathogen capture

A feasible approach for detecting rare analytes is immuno-capture or aptamer trapping, wherein immobilized antibodies or aptamers are used for an initial enrichment step 42, 43. To detect proteins and other small molecules, aptamer trapping can be used for target capture before LC-MS or other analyses 42, 44. Aptamer-mediated pathogen capture is frequently combined with PCR or other nucleic acid amplification technologies to increase detection sensitivity. Because nucleic acid aptamers can be PCR-amplified, two PCR amplification strategies that use either nucleic acids of analyte (microorganisms) or aptamers as PCR templates can be considered for detection.

For protozoa and their eggs, bacteria and their spores, fungi and their spores, and DNA virus particles, real-time quantitative PCR (qPCR) can be used for detection. qPCR can be performed directly using the DNA of the enriched microorganisms. This strategy has been used to detect Bacillus cereus spores 43, Alicyclobacillus spores 45, Salmonella 46, 47, Escherichia coli 47, Campylobacter jejuni 48, and Listeria spp 49. For RNA viruses and other nucleic acid-free targets, an alternative strategy using one immobilized aptamer or antibody for target capture and another detection aptamer for PCR templates can be considered. Compared to direct PCR detection for pathogens, aptamer-mediated pathogen capture increases the detection sensitivity due to the pathogen enrichment by aptamers. However, PCR amplification using nucleic acid of pathogens as templates for detection is less sensitive and time-consuming due to the procedures of DNA/RNA isolation and thermocycling machine-required.

Nucleic acid amplification using aptamers as templates is more sensitive and convenient than that using pathogens' nucleic acid as templates. Aptamer-combined isothermal nucleic acid amplification techniques have been broadly used to facilitate pathogen detection. The rolling circle amplification (RCA) technique uses a single-stranded DNA probe derived from circular DNA formed by ligation when the analyzed sequence is complemented with the DNA probe at both terminals 50. This formes single-strand circular DNA hybridizes with the primer to generate a sequence consisting of numerous copies of analyte DNA in the presence of phi29 or Bst DNA polymerase under homothermal conditions. Song et al. used aptamer-combined RCA to detect Vibrio parahaemolyticus 51. In detail, they used two V. parahaemolyticus-specific aptamers as probes. The first probe was a biotin-labeled aptamer for bacteria capture, and the second was a sequence containing a recognition site for Nb.BbvCI and Nb.Btsl at the 5'- and 3'-terminals, respectively, and a sequence encoding a G4 structure in products at the 3'-terminal. When these two probes were bound to bacteria, probe 2 could be enriched and collected through probe 1 and streptavidin-conjugated beads. Thus, the amount of collected probe 2 indicated the number of bacteria in the tested sample. Subsequently, the collected probe 2 was seamed to form a circular DNA under the assistance of a splint sequence and T4 ligase, followed by an RCA assay, producing numerous copies of G4 structure that then complexed with hemin molecules to catalyze the oxidation of ABTS^2-^ to ABTS^• -^, generating a green color observed by the naked eye. In addition to RCA, strand-displacement amplification (SDA) based on aptamers was developed for pathogen detection. Cai et al. developed an aptamer-based SDA assay to detect Staphylococcus aureus 52. They immobilized an S. aureus-specific aptamer that linked a partially complementary ssDNA of the aptamer to a magnetic bead. Upon binding with S. aureus, the complementary ssDNA was released from the beads, resulting in a direct link between the amount of dissociative ssDNA and S. aureus. The amount of dissociative ssDNA was amplified by the following SDA and measured by fluorometry. The above designs can be applied to detect other pathogens or pathogen components after developing specific aptamers.

Because aptamer-combined nucleic acid amplification is based on aptamer-based target capture, the binding specificity and affinity of the capture aptamer must be prioritized to avoid false positives and being affected by the immobilization and addition of other nucleic acids, such as G4 structures. When using two target-specific aptamers, the aptamers must bind to different sites of the same target and should not mutually affect target binding. Although several different aptamers could be developed in one round of SELEX, two aptamers for aptamer-based nucleic acid amplification may result from various rounds of SELEX. The multivalent aptamer isolation SELEX (MAI-SELEX) established by Gong et al. may be useful 53. Because nucleic acid aptamers can be amplified, a well-designed aptamer-based amplification assay for pathogen detection is more sensitive and convenient than an antibody-based detection method.

### Aptamers applied for pathogen detection

#### Viruses

Viruses are obligate intracellular parasites with a diameter of 20-300 nm. Generally, viral particles (virions) consist of a DNA or RNA genome and a genome-protective protein coat (capsid). Despite their simple structure, viruses can cause a variety of diseases in animals and plants. The World Health Organization (WHO) reports that over 15 pandemic or epidemic diseases, including the current COVID-19 pandemic, are caused by viruses. Early detection is a crucial stage of response intervention for epidemics and allows rapid implementation of containment measures to reduce the risk of amplification and potential international spread 54. Relevantly, aptamer-based technology was broadly developed for the detection of virus particles or virus-contaminated samples from humans, livestock, fish, and even plants. Indeed, more than 150 nucleic acid aptamers targeting 34 virus species, including those viruses in the WHO's list of pandemic or epidemic diseases have been developed (Table 1, detailed in Supplementary material S1). Most of these aptamers were selected using viral surface proteins as targets. However, the structures of recombinant proteins are not always identical to natural structures. Accordingly, aptamers selected using recombinant proteins cannot bind to their natural targets well. Other aptamers were selected using whole viral particles via nitrocellulose membrane or by immobilizing virus particles on microbeads. Since lots of aptamers targeted various viruses were developed, we summarized those aptamers and aptamer-based detection examples separately, according to species of virus, in this section. One can use these aptamers to develop detection methods directly or improve these established methods to make detection methods more reliable and sensitive.

##### Influenza virus

Hemagglutinin (HA) proteins have been used for aptamer selection for influenza A and B viruses. Using full-length glycosylated recombinant HA protein as the target, Wang and colleagues selected a 73-mer DNA aptamer with an equilibrium dissociation constant (K_d_) of 4.65 nM 71. This aptamer was further developed for influenza A virus (IAV) H5N1 detection using QCM, SPR, or electronic aptasensor techniques 29, 31, 72-74. The detection limit can be as low as 2^-4^ hemagglutination units (HAU)/50 uL, suggesting that this aptamer is reliable for the development of a detection method for the IAV H5N1 virus. Likewise, other DNA or RNA aptamers against HA proteins were developed, including H1N1 75-77, H3N2 78, H5N1 63, 64, 78, and IBV 79. According to the reported sequences of those aptamers against HA proteins, we summarized the character of those sequences as “5'-GGT GNG CAR GAN RNN GTG KSN NNN NRN NNN NNG GCA CAN SSG T-3'”.

Aptamers targeting IAV can also be selected using the whole viral particle as well. Lai and colleagues immobilized the whole H1N1viral particle (positive selection) or IBV (negative selection) on magnetic beads using IAV- or IBV-specific antibodies to form an integrated SELEX microfluidic chip, resulting in a 72-mer DNA aptamer with a K_d_ of 55.14 nM 61. Based on this aptamer, IAV can be detected using aptamer-trapping PCR and a fluorescent probe, with detection limits of 0.0064 and 0.032 HAU, respectively 61, 62. Using the graphene-oxide-based SELEX procedure, Kim and colleagues developed two H5N2 virus-specific DNA aptamers which were then used to detect the H5N2 virus using lateral flow strips, with a limit of 1.27×10^5^ EID_50_/mL in the buffer and 2.09×10^5^ EID_50_/mL in the duck's feces, respectively 80.

The above reports indicate that protein-SELEX was more popular than virus particle-SELEX for IAV-specific aptamer selection and aptasensors were much sensitive for IAV detection. All these selected aptamers enriched the aptamer resource for IAV detection using aptamer-based techniques.

##### Human immunodeficiency virus (HIV)-1

In 2000, Yamamoto et al. developed an RNA aptamer, RNA^Tat^, targeting Tat proteins or peptides derived from either HIV-1 or HIV-2 81. This aptamer was recently used to detect HIV-1 using an unmodified GNP-based colorimetric assay and split RNA^Tat^ 82. The detection limit of Tat protein was 10 nM. Based on RNA^Tat^, a spectrophotometric ellipsometry-based aptasensor was developed to detect Tat protein with a detection limit of 1 pM 83. In addition, a 38-mer DNA aptamer binding to the reverse transcriptase of HIV-1 was also developed 84, 85 and eventually used for HIV-1 detection via radioactivity-based reverse transcriptase nucleotide incorporation assays, with a detection limit of 100-300 virions 60.

##### Hepatitis virus

Using the E antigen of hepatitis B virus (HBV) as the target, DNA aptamers were developed and applied for the detection of the E antigen with detection limits of the enzyme-linked oligonucleotide assay and the fluorescence-based aptasensor were 0.1 ng/mL 58, 86 and 609 ng/mL 87, respectively. An aptamer targeting the surface antigen of HBV (HBsAg) was also developed for HBV detection using a chemiluminescence aptasensor technique, with a detection limit of 0.1 ng/mL 88. Similarly, the core antigen of the hepatitis C virus (HCV) was used as a SELEX target to develop a 59-mer RNA aptamer and seven DNA aptamers 89, 90. The RNA aptamer was used to detect HCV core antigen via lateral flow strip technology, with a detection limit of 10 pg/mL 59. One of the resulting DNA aptamers was combined with electrochemical impedance spectroscopy to detect HCV core antigen, with a detection limit of 3.3 pg/mL 91.

##### Severe acute respiratory syndrome (SARS) virus

The outbreak of SARS caused by the SARS-CoV virus in 2002 affected thousands of people. Currently, the continuing COVID-19 pandemic caused by SARS-CoV-2 affects tens of millions of people worldwide. Consequently, scientists in various fields around the world are collaborating to help end the pandemic. Detection technologies for SARS-CoV-2 have been systematically summarized 92, 93. Some of these techniques can be performed based on aptamers 94, but only after sufficient aptamers against SARS-CoV-2 are developed. Before the COVID-19 pandemic, RNA and DNA aptamers targeting nucleocapsid (N) protein or NTPase/helicase of the SARS-CoV virus were developed. Jang et al. established an RNA aptamer pool against the SARS-CoV NTPase/helicase containing an AG-rich conserved sequence of 10-11 nucleotides [AAAGGR(G)GAAG; R, purine base] and/or an additional sequence of 5 nucleotides [GAAAG] 95. This pool may be useful in screening SARS-CoV-specific RNA aptamers for SARS-CoV detection. Ahn et al. targeted the recombinant N protein of SARS-CoV to develop RNA aptamers using SELEX, producing a 92-mer aptamer 65. Using a chemiluminescence immunosorbent assay and a nanoarray aptamer chip, they detected N protein at concentrations as low as 2 pg/mL. Further, this aptamer was used in an optical QD-based RNA aptamer chip to detect the N protein of SARS-CoV, with a detection limit of 0.1 pg/mL 66. Moreover, a 112-mer DNA aptamer against the N protein of SARS-CoV with a K_d_ of 4.93±0.30 nM was also developed 96. Using this DNA aptamer, N protein can be detected via western blot. After the COVID-19 outbreak, this DNA aptamer was truncated and tested for binding to SARS-CoV-2 N protein 97. Besides, Zhang et al. developed 4 DNA aptamers by SELEX against SARS-CoV-2 N protein with high binding affinities 98. They further used two of these aptamers (A48 and A58) to detect N protein via ELONA whose detection limit was 20 pM. Subsequently, Liu et al. employed A48 and A58 aptamers to detect serum SARS-CoV-2 N protein by the aptamer-assisted proximity ligation assay, with a detection limit of 37.5 pg/mL 99.

Recently, two novel DNA aptamers against the spike glycoprotein (S) of SARS-CoV-2 were developed with K_d_ values of 5.8 nM and 19.9 nM 100. One of the aptamers was applied to detect SARS-CoV-2 using a surface-enhanced Raman scattering (SERS)-based aptasensor with a detection limit of 10 PFU/mL 101. Briefly, the aptamer was labeled with Cy3 and immobilized by an oligo T_15_ linker complemented with a nanogold-immobilized oligo A_15_. Upon binding to S protein, aptamers deviate from the nanogold, resulting in a SERS peak intensity change that can be monitored. Cennamo et al. immobilized one of the aptamers on a short polyethylene glycol (PEG) interface on gold nanofilm deposited on a D-shaped plastic optical fiber (POFs) probe to form an SPR aptasensor to detect S protein with a detection limit of 37 nM. These designs were mechanically simple but ivolved sophisticated instruments.

Interestingly, other detection methods did not depend on virus-specific aptamers but were developed based on fluorescence dye-specific aptamers. For instance, Woo et al. established a sensitive SARS-CoV-2 detection assay based on the fluorescent dye RNA aptamer and one-pot isothermal ligation and transcription technologies, which can be performed within 30-50 min of incubation time and can reach a limit of detection of 0.1 aM RNA concentration 102. In detail, they linked the T7 promoter with an ssDNA probe that hybridizes with the target SARS-CoV-2 RNA and linked the template ssDNA for a dye-binding RNA aptamer with another ssDNA probe that contains the complementary sequence to the other half of the target RNA region. When the two probes hybridize with the target, the SplintR ligase would ligate the two probes and then, the RNA aptamer would be transcribed by the T7 RNA polymerase from the ligation product and bind with the fluorescent dye to induce the detectable fluorescence. Another fluorescent dye-specific RNA aptamer, broccoli, was combined with CRISPR-Cas13 technology to detect SARS-CoV‑2 and its mutated variants with a detection limit of 82 copies 103. A split couple of DNA probes that specifically recognize viral RNA were designed. After binding to viral RNA, the probes come together and are ligated by a T4 ligase 2 under the guidance of the promoter sequence anchored in one of the probes, yielding a template for in vitro transcription. The produced template is then transcribed to RNA, which is recognized by CRISPR RNA (crRNA) to recruit and activate Cas13a proteins, leading to broccoli RNA aptamer degradation and diminished fluorescence in the detection mixture. As there is no need for new aptamer selection, virus-specific aptamer-independent detection methods are more applicable and should be further developed for emergent viruses detection, including SARS-CoV-2.

**Figure 5:**
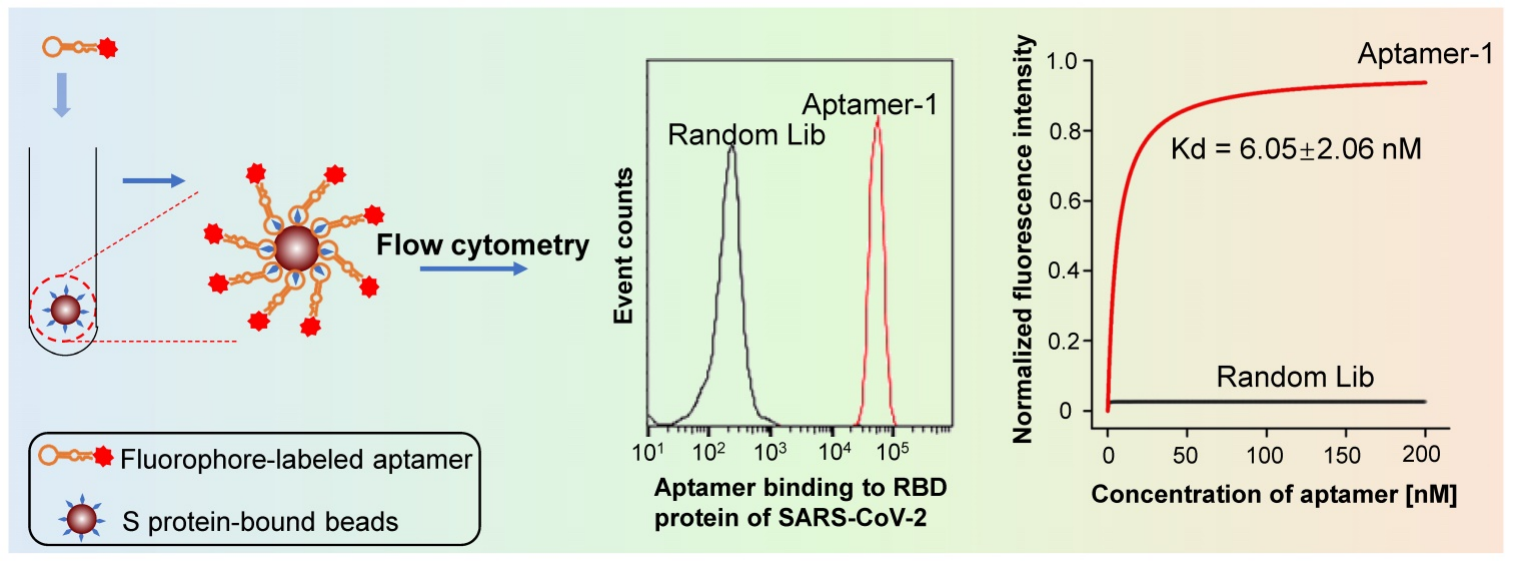
Flow cytometry analysis reveals that the aptamer developed in our lab binds the RBD of S protein of the SARS-CoV-2 virus with high affinity. In contrast, a random sequence library showed no binding under the same experimental conditions.

We developed SARS-CoV-2-specific DNA aptamers as well. Our functional study reveals that t binds to either S or the receptor-binding domain (RBD) with high affinity (K_d_ = 6.05 nM) (Figure 5) 67. Furthermore, our findings indicate that the aptamer can be used to detect living viral particles, using ddPCR technology (the detection method is establishing).

##### Dengue virus (DENV)

Dengue caused by mosquito-borne DENV is endemic in more than 100 countries. According to data from WHO, 4.2 million dengue disease cases were reported in 2019. Aptamers targeting DENV have been broadly developed. Several aptamers against recombinant envelope antigens of four serotypes of DENV were identified by Bruno and colleagues 104. A 41-mer DNA aptamer targeting the envelope antigen domain III was developed 105 and applied to detect DENV using a GNP-based colorimetric assay 106. According to the reported sequences of those aptamers against envelope antigen of DENV, we summarized the character of those sequences as “5'-RSR SKR YRN NNS GNN TSC NNG NRS NNN GGN NK-3'”. Besides the envelope antigen, other proteins of DENV were used as the target for aptamer selection. Jung et al. identified 2'-fluoro-modified RNA aptamers that can specifically bind DENV serotype 2 (DENV2) methyltransferase using SELEX 107. Not only aptamer-targeting proteins of DENV but also RNA aptamers binding to RNA elements, such as 5'-UTR of DENV have been identified 108. Although some of the aptamers were not further exploited to detect DENV, these aptamers should be used to establish simple and rapid detection approaches for DENV.

##### Ebola virus

Ebola virus infects humans and other primates to cause a severe, often fatal illness named Ebola virus disease or Ebola hemorrhagic fever. A 2′-fluoro pyrimidine-modified RNA aptamer was found to specifically bind a soluble glycoprotein of the Ebola virus with a K_d_ of 54 nM 109. Recently, Hong et al. established an aptamer selection platform based on magnetism-controlled selection chips 56. Using this platform, they developed several DNA aptamers with high binding affinities against glycoproteins and nucleoproteins of the Ebola virus. Furthermore, based on the binding of these aptamers with glycoprotein, they contrived an applicable Ebola virus-detection system with a detection limit of 4.2 ng/mL. These studies provide important materials for future Ebola virus detection applications.

##### Norovirus

Noroviruses also referred to as winter vomiting bugs are the foremost cause of gastroenteritis outbreaks throughout the world. Aptamer-based detection methods have been studied by a few teams. Giamberardino et al. developed a 57-mer DNA aptamer that binds to murine and human norovirus using SELEX against the whole viral particle 110. This aptamer was applied as an electrochemical sensor using a GNP-modified screen-printed carbon electrode or colorimetric NanoZyme aptasensor, with detection limits of 180 and 30 virus particles/mL, respectively 110, 111. Escudero-Abarca et al. also used the whole viral particle as the target for aptamer selection to develop a 40-mer DNA aptamer 112. Using this aptamer as a capture trap or probe combined with qPCR or fluorometry, human norovirus was detected with a detection limit of 10 RNA copies in lettuce samples or 80 ng/mL in water, respectively 112, 113. Chand and Neethirajan established a microfluidic platform integrated with a graphene-gold nanocomposite aptasensor based on an 81-mer DNA aptamer for norovirus detection, with a detection limit of 100 pM in blood samples 114.

##### Other human viruses

In 2012, Bruno et al. reported numerous DNA aptamers against arbovirus including Chikungunya, Crimean-Congo hemorrhagic fever, dengue, tick-borne encephalitis, and West Nile virus discovered using enzyme-linked aptamer sorbent assays as the aptamer screening method 104. These published DNA aptamer sequences provide a valuable resource for future arbovirus detection endeavors. A 72-mer DNA aptamer targeting VP1 structural polypeptide of foot-and-mouth disease virus was selected and further developed for virus detection using a competitive FRET assay, with a detection limit of 25 ng/mL 57. To detect respiratory syncytial virus, Percze et al. developed a series of DNA aptamers against the antibody-immobilized inactivated virus 35. These aptamers were conjugated with fluorophores and applied as probes for respiratory syncytial virus detection with a limitation of a single virus particle in throat swab samples 115. The severe fever with thrombocytopenia syndrome (SFTS) virus was targeted by a 40-mer DNA aptamer developed against the SFTS nucleocapsid protein (NP). Combined with antibodies, this aptamer was further developed to detect NP of SFTS virus using ELONA, with a detection limit of 0.009 ng/mL 68.

Zika virus is a mosquito-borne flavivirus that can cause preterm birth or miscarriage in women during pregnancy. Lee and Zeng developed two DNA aptamers against non-structural protein 1 (NS1) of the Zika virus using conventional SELEX 69. Using an aptamer-based ELISA assay, the 5'-amino C6-modified 41-mer aptamer was immobilized onto an amine-reactive maleic anhydride-activated plate for virus capture, with antibodies or another 5'-biotinylated aptamer used for virus detection 69. The detection limit of the aptamer-aptamer model was 100 ng/mL, while the aptamer-antibody model was 0.1 ng/mL, suggesting that the aptamer-antibody combination is a promising strategy for virus detection, especially for viruses with limited available antibodies.

##### Animal and plant viruses

Viral diseases adversely affect animal husbandry and agriculture. An economical and applicable pathogen detection system is urgently needed. Several aptamer-based detection methods for animal and plant viruses have been developed. Lu et al. developed a 49-mer DNA aptamer against Muscovy duck parvovirus (MDPV) with a K_d_ of 467 nM after 15 rounds of SELEX 55. Using this aptamer, they employed a GNP-based colorimetric assay to detect MDPV with a detection limit of 1.5 EID_50_ (50% egg infection dose) or 3 EID_50_ for measurements using spectrophotometry or the naked eye, respectively. To detect the Newcastle avian virus (NAV), a series of DNA aptamers were selected using SELEX against the whole NAV. Two of these were further applied for NAV detection using ELONA with a detection limit of 1.2 EID_50_/mL 70. Aptamers were also developed for aquatic animal viruses detection. Ye et al. obtained three DNA aptamers against grass carp reovirus (GCRV) infected cells using cell-SELEX, which may be applied in the development of rapid diagnosis technology and antiviral agents against GCRV infection in aquaculture 116. To detect the red-spotted grouper nervous necrosis virus (RGNNV), two therapeutic DNA aptamers against the coat protein of RGNNV were used to form a lateral flow assay 117. Briefly, one of the aptamers was modified with biotin for virus enrichment using streptavidin-conjugated magnetic beads, resulting in the enrichment of another RGNNV-specific aptamer for isothermal strand displacement amplification. Amplification products were detected using a lateral flow assay with nanogold particles. Additional aptamers against aquatic animal viruses have been reviewed previously 118 and are useful for animal disease diagnosis and therapy. Aptamers against economic plant viruses have also been developed. Lautner et al. developed two aptamers targeted to the apple stem pitting virus (ASPV) using the SPR system or ELONA 119, 120. To prevent the outbreak of animal and plant diseases, sensitive and convenient monitoring systems for pathogens should be developed and installed on farms. In this regard, aptamers against various animal and plant viruses hold broad application prospects.

#### Bacteria

Bacteria are microscopic, single-celled organisms that exist in millions of environments, both inside and outside other organisms. Though some bacteria are harmful, others serve useful purposes. Harmful bacteria that cause bacterial infections and diseases are termed pathogenic. Bacterial diseases occur when pathogenic bacteria get into the body, reproduce, and crowd out healthy bacteria, or replicate in tissues that are normally sterile. Harmful bacteria may also emit damaging toxins. Because of their sufficient size, bacteria can be harvested using a centrifuge and most aptamers against bacteria can be selected with whole-cell-based SELEX. Representative research is summarized below (Table 2). More detailed information regarding these aptamers is summarized in Supplementary material S1.

##### Escherichia coli

*E. coli* is a type of bacteria that normally lives in the intestines of humans and other animals. Most types of *E. coli* are harmless and even help to keep the digestive tract healthy, but some strains can cause diarrhea. *E. coli* O157:H7 is a very threatening strain that causes abdominal cramps, vomiting, bloody diarrhea, and even acute kidney failure in children. Several DNA aptamers targeting *E. coli* have been developed using lipopolysaccharide (LPS) or whole-cell O157:H7 as the target. Using the US patent-originated aptamers 144, Wu et al. established an aptamer-based colorimetric detection method for O157:H7 using truncated DNA aptamers against LPS, with a detection limit of 10,000 CFU/mL 145. Based on these aptamers, electrochemical aptasensor and lateral flow strip assay for O157:H7 detection were invented 146, 147, lowering the detection limit to 10 CFU/mL. These reports indicate that these aptamers published in the US patent can be further applied for the development of commercial products. Another 45-mer DNA aptamer targeting whole-cell O157:H7 was developed using cell-SELEX and used for detection via QCM-sensor technology, with a detection limit of 1460 CFU/mL 124. A 72-mer DNA aptamer was immobilized on magnetic beads to capture O157:H7 in blood samples for detection 148. The same DNA aptamer was used in a hydrothermally grown ZnO nanowire array to construct a high-performance photoelectrochemical aptasensor to detect O157:H7, with a detection limit of 1.125 CFU/mL 149. Comparing to clinic detection methods, these aptamer-based detection techniques reduced the detection time and increased the detection sensitivity.

In 2008, Bruno et al. developed DNA aptamers against LPS from *E. coli* O111:B4 using SELEX against LPS-conjugated magnetic beads 150. Subsequently, some of these aptamers were used to detect *E. coli* LPS. Xie et al. developed an aptasensor based on a hybridization chain reaction for LPS detection with a detection limit of 1.73 ng/mL 151. Zhu et al. employed a dual aptamer-functionalized GNP probe to detect LPS, reaching a detection limit of 1 μg/mL 152. DNA aptamers targeting other features of *E. coli* were developed and broadly applied for *E. coli* detection. Bruno et al. described a high-throughput DNA aptamer screening and detection method based on competitive FRET technology 123. With this method, they obtained several DNA aptamers targeting the outer membrane proteins of *E. coli* 8739 and established an *E. coli* 8739 detection method with a detection limit of 30 CFU/mL in binding buffer solution. DNA aptamers against the fimbriae protein of enterotoxigenic *E. coli* K88 were developed with a K_d_ of 25 nM and may be further applied for *E. coli* K88 detection 153.

Using whole cells of *E. coli* as the SELEX target, Kim et al. isolated 28 DNA aptamers that bind to whole-cell *E. coli*. Among these, four were further characterized with K_d_ values ranging from 12.4 to 25.2 nM 154. These aptamers were combined as a cocktail to detect *E. coli* with a detection limit of 370 CFU/mL 155. Using the same aptamers, Jin et al. developed a detection method based on FRET aptasensor technology and reached a detection limit of 3 CFU/mL 156. Likewise, Hua et al. used the same aptamers to construct a sensitive potentiometric resolved ratiometric photoelectrochemical aptasensor for *E. coli* detection with a detection limit of 2.9 CFU/mL 157.

##### Salmonella

Salmonella is the type of bacteria most frequently reported as causing food-related illness in the United States. People and animals can carry salmonella in their intestines and feces. The bacteria often spread through contaminated foods. As such, rapid and sensitive detection is of critical importance. Joshi et al. selected a DNA aptamer targeting the outer membrane proteins of *S. enterica* serotype Typhimurium and obtained five aptamer candidates. Using one of the aptamers (5'-TAT GGC GGC GTC ACC CGA CGG GGA CTT GAC ATT ATG ACA G-3'), they developed an aptamer-based trapping PCR detection method with a detection limit of 1 CFU/mL in rinsate samples 46. This aptamer was broadly applied in various detection methods based on different aptasensor technologies, with detection limits ranging from 1 to 1000 CFU/mL 47, 158-168, suggesting that this aptamer is worth to be applied to develop commercial products. In addition, using Vi-antigen as the target, Pathania et al. developed a DNA aptamer using a microtiter-based SELEX approach. This aptamer was applied as an electrochemical aptasensor for Vi-antigen detection with a limit of 100 pg/mL 134.

Since bacteria can be collected by centrifuge, many aptamers have been developed using cell-SELEX. For example, Duan et al. developed a DNA aptamer using cell-SELEX and immobilized this aptamer on magnetic beads for *S. Typhimurium* capture. Then, the bead-captured *S. Typhimurium* could be detected by the FAM-labeled aptamer using a fluorescence spectrophotometer. The detection limit of this aptamer-based fluorescent assay for *S. typhimurium* was 25 CFU/mL 169. This design is quite simple and even can be used to detect *S. typhimurium* by using a magnet and a fluorescent excitation lamp. However, due to the same aptamer was both used to capture and fluorescent development, there is a binding competition between the immobilized aptamer and the fluorophore-labeled aptamer, resulting in low sensitivity in detection. Therefore, two different aptamers used in this design should improve the detection sensitivity and specificity. Besides, if the detection aptamer can be labeled with other dyes that can be observed by the naked eye, the detection assay should be more convenient. Another example, Wang et al. obtained a 90-mer DNA aptamer using QCM-based SELEX and developed a QCM-based aptasensor for *S. Typhimurium* detection with a limit of 1000 CFU/mL 170. To improve the detection sensitivity, they further applied this aptamer for *S. Typhimurium* detection using aptamer-based PCR and electrochemical impedance aptasensor technologies 135, 171, lowering the detection limit to 100 CFM/mL and 80 CFU/mL, respectively. A series of high-binding affinity DNA aptamers against the whole bacterial cells of *S. enteritidis* and *S. typhimurium* were developed 172, 173. Later, those aptamers were used in a fluorometric GO-based assay for *S. enteritidis* detection with a limit of 25 CFU/mL 39 and a fluorescence-based assay for *S. typhimurium* detection with a limit of 20 CFU/mL 173.

##### Mycobacterium tuberculosis

*M. tuberculosis* is the causative agent of tuberculosis, one of the top 10 causes of death globally. Aptamers targeted to different proteins of *M. tuberculosis* were developed. Qin et al. developed DNA aptamers against the MPT64 antigen 174. These aptamers were then applied for an electrochemical aptasensor for ultrasensitive detection of *M. tuberculosis* with a limit of 20 fg/mL in human serum samples 175. Ansari et al. identified an 80-mer aptamer that binds to the FbpA protein of *M. tuberculosis* with high affinity using protein-SELEX 127. They further applied this aptamer in a GO-based fluorometric assay for FbpA detection, with a limit of 2.1 nM in human serum samples. Dhiman et al. developed a 28-mer DNA aptamer against the biomarker of *M. tuberculosis*, HspX antigen, using protein-SELEX 128. This aptamer was used for *M. tuberculosis* infection diagnosis via ELONA and electrochemical sensor systems, with a sensitivity as high as ELISA and qPCR, but with a lower cost 129, 130, 176.

##### Staphylococcus

*S. aureus* is a commensal organism that resides in the skin and mucosa with a major impact on human health. It generally causes skin and soft-tissue infections but can infect other organ systems to cause life-threatening diseases as well 177. Chang et al. screened two 62-mer DNA aptamers using SELEX against the whole-cell of *S. aureus* and further developed a rapid detection method that can detect one single bacterium based on GNP-related technology 178. These two aptamers were further applied to the development of other detection methods for *S. aureus*, based on silver nanoparticles or an aptamer-based magnetic beads capture platform 148, 159. The detection limits of those methods ranged from 1-10 CFU/mL. Using the whole-cell as the SELEX target, Cao et al. developed five DNA aptamers and found that the combined aptamers can probe single bacterium *S. aureus* in pyogenic fluid samples 179. These aptamers have since been broadly applied in the development of *S. aureus* detection methods. For example, Duan et al. used one of the aptamers to form aptamer-functionalized magnetic nanoparticles for *S. aureus* detection with a limit of 8 CFU/mL 158. Three of the aptamers were used to detect *S. aureus* infection *in vivo* and *ex vivo* via technetium-99m radiolabeling 180. These aptamers have been used in aptasensors 52, 138, 139, 181-183, probes and trapping 184-187, and ELONA assays 188 to enable *in vitro S. aureus* detection. The detection limits of these methods ranged from 1 to 35 CFU/mL. Those published works indicate that these five aptamers are reliable and worthy to be applied to develop commercial products for the detection of *S. aureus*. Sheng et al. combined one of the aptamers, SA31, with ssDNA transcribed to the broccoli aptamer to develop a transcription aptasensor for *S. aureus* detection with a detection limit of 77 CFU/mL 189. They assembled the broccoli ssDNA with a promoter and binder ssDNA to form L-broccoli. The SA31 aptamer was initially blocked by its complementary ssDNA. Upon SA31 aptamer binding to *S. aureus*, the blocker ssDNA shed from SA31 and hybridized with the binder ssDNA, resulting in the extension of L-broccoli and exposing the template for broccoli RNA aptamers. The produced broccoli aptamer bound to fluorogenic DFHBI-1T to activate fluorescence with the fluorescent intensity indicating the amount of *S. aureus* in samples.

Other research groups developed aptamers against the surface proteins of or exotoxins secreted by *S. aureus* that are responsible for food poisoning. To detect methicillin-resistant *S. aureus*, Qiao et al. combined a penicillin-binding protein 2a (PBP2a)-specific aptamer with an *S. aureus* antibody to establish a fluorometric assay with a detection limit of < 1380 CFU/mL in spiked nasal swab samples 190. A DNA aptamer against protein A of *S. aureus* developed using SELEX and applied to ELONA or impedimetric aptasensor technology, with a detection limit of 10 CFU/mL 191-193.

##### Vibrio

Some *Vibrio* species cause vibriosis and an estimated 80,000 illnesses and 100 deaths in the United States alone every year. The most common species causing human illness in the United States are *V. parahaemolyticus*, *Vibrio vulnificus*, and *Vibrio alginolyticus*. The presence of *Vibrio* bacteria in the stool, wound, or blood of a patient with vibriosis symptoms indicates infection. Several aptamers targeting *V. parahaemolyticus*, *V. vulnificus*, and *Vibrio fischeri* have been developed.

Duan et al. developed a series of DNA aptamers binding to *V. parahaemolyticus* using whole-cell SELEX 194. To detect *V. parahaemolyticus*, this group used a QD-conjugated aptamer combining its complementary ssDNA as the aptasensor, or an aptamer-functionalized chromatophore as the capture probe. The detection limits of these methods were 5000, 35, and 15 CFU/mL, respectively 158, 195, 196. Using aptamer-immobilized magnetic beads as a detection vehicle, Wu et al. developed optical aptasensors that improved the detection limit to 10 CFU/mL 142, 181. Interestingly, Sun et al. used combined this aptamer with trivalent G4 DNAzyme, hemin, and magnetic beads to form a colorimetric aptasensor that could visibly detect *V. parahaemolyticus* at concentrations as low as 10 CFU/mL in food samples 141. In detail, they immobilized the biotinylated aptamer on streptavidin beads, then bound the trivalent G4 DNAzyme-linked aptamer-complementary ssDNA with the immobilized aptamers. When the immobilized aptamers bind to their targets, the ssDNA is released into the supernatant due to structural change of the aptamers, resulting in that the supernatant contains the trivalent G4 DNAzyme which can change the color of TMB through catalyzing H_2_O_2_. This design is promising for commercialization because it is simple to operate and does not rely on precise detection machines. The key of this design is to ensure the high binding affinity of immobilized aptamers to their targets and the release of the ssDNA from aptamers upon aptamer-target binding.

Using whole-cell SELEX, Yan et al. and Shin et al. developed DNA aptamers targeting *V. vulnificus* and *V. fischeri*, respectively 140, 143. The *V. vulnificus-*targeted aptamer was used as a trap for the following PCR detection assay, the detection limit of which was 8 CFU/mL 143. Using the two *V. fischeri*-targeted aptamers and GNPs, a lateral flow strip for *V. fischeri* detection with a limit of 40 CFU/mL was established. Liu et al. also developed two DNA aptamers targeting *V. vulnificus* using whole-cell SELEX and applied them as probes for *V. vulnificus* detection using flow cytometry, with a detection limit of 29.96 CFU/mL 197.

##### Other bacteria

Aptamers targeting other pathogenic bacteria have also been developed. Dwivedi et al. developed an 80-mer DNA aptamer against *Campylobacter jejuni* using whole-cell SELEX 198. This aptamer was further applied as a colorimetric aptasensor for *C. Jejuni* detection, with a detection limit of 100 CFU/mL 122. Lamont et al. combined a series of DNA aptamers targeting *Francisella tularensis* as an aptamer cocktail to enrich *F. tularensis* in foods and environmental matrices 199. Bitaraf et al. characterized DNA aptamers targeting *Haemophilus influenzae* type b 200. Using a fluorescently labeled aptamer as a probe and flow cytometry, *H. influenzae* can be detected in the cerebrospinal fluid of patients with a detection limit of 60 CFU/mL.

Several aptamers against *Listeria*, the causative agent of listeriosis, have been developed. Using whole-cell SELEX, Duan et al. identified a 35-mer DNA aptamer targeting *L. monocytogenes* with a K_d_ of 48.74 nM. Additionally, using this aptamer, they developed a sandwich-type fluorescent bioassay for *L. monocytogenes* detection, with a limit of 75 CFU/mL 126. Suh et al. developed a DNA aptamer targeting *L. monocytogenes* using whole-cell SELEX and further applied this aptamer as a trap to capture *L. monocytogenes*, with a detection limit of 120 CFU/mL 49. Later, Suh et al. again performed whole-cell SELEX and identified ten DNA aptamers that bound to *L. monocytogenes* at different growth phases 201. When combined with the *L. monocytogenes*-specific antibody as a capture agent, these probe aptamers were able to detect *L. monocytogenes* via qPCR 125. Protein-SELEX for *L. monocytogenes* has also been performed. Ohk et al. developed DNA aptamers targeting either the Lmo0610 or internalin A protein of *L. monocytogenes* 202. These aptamers were further applied as fluorescent probes or potentiometric aptasensors for *L. monocytogenes* detection, with a detection limit of 1000 CFU/mL in food samples and 10 CFU/mL in environment samples, respectively 202, 203.

Aptamers against other bacteria, including Bifidobacterium bifidum 121, Brucella melitensis 30, Neisseria meningitidis 131, Porphyromonas gingivalis 204, Proteus mirabilis 205, Pseudomonas aeruginosa 206, Shigella dysenteriae 207, Shigella sonnei 208, and Treponema denticola 204 have been developed using whole-cell SELEX. Aptamers targeting bacterial spores from Alicyclobacillus 45, Bacillus anthracis 209, and Bacillus cereus 43 have also been described. Aptamers targeting parasitic mycoplasma were developed using SELEX against bacterium-infected cells 210; some of these were further used to detect bacteria in various samples using ELONA 128, aptasensor 30, probe 36, or aptamer-based trapping technologies 43. Interestingly, aptamers targeting the outer membrane vesicles (OMVs) of Gram-negative bacteria were developed for OMV detection using ELONA technology 211, 212. These aptamers laid the foundation for the subsequent development of diagnostic methods for those pathogenic bacteria in food, water, and clinical samples.

#### Fungi

There are millions of fungal species, but only a few hundred can cause human illness. Fungal infections are common throughout much of the natural world. In humans, fungal infections occur when an invading fungus takes over an area of the body and overwhelms the immune system. Compared to viral and bacterial infections, fungal infection is uncommon and unlikely to be life-threatening. The CDC divides fungal diseases into four types: (1) common fungal diseases, including fungal nail infection, vaginal candidiasis, ringworm, and *Candida* infections of the mouth, throat, and esophagus; (2) fungal diseases that affect people who live in or travel to certain areas, including blastomycosis, coccidioidomycosis or valley fever, *Cryptococcus gattii* infection, histoplasmosis, and paracoccidioidomycosis; (3) fungal diseases that affect people with weakened immune systems, including aspergillosis, *Cryptococcus neoformans* infection, *Candida auris* infection, mucormycosis, *Pneumocystis* pneumonia, and *talaromycosis*; (4) other fungal diseases, including fungal eye infection, mycetoma, and sporotrichosis.

To detect fungal diseases, aptamers have largely been developed against mycotoxins, which are toxic secondary metabolites produced by fungi. Based on these aptamers, many promising aptasensors have been developed for the detection of various mycotoxins 213-215. Detection of mycotoxin contaminants, and pathogenic fungi are important for public health. Nucleotide aptamers targeting infectious fungi have also been developed. Tang et al. developed two high-affinity DNA aptamers specifically targeting (1→3)-β-D-glucans from the cell wall of *Candida albicans* using SELEX 216. These two aptamers were used to establish an ELONA assay for (1→3)-β-D-glucans detection in serum samples.

#### Parasites

Parasites are organisms that live off other organisms (hosts) to survive. Some parasites can make their hosts sick, resulting in parasitic diseases 217. Parasitic infections are common in tropical and subtropical regions of the world. There are three main classes of parasites that cause disease in humans: protozoa, helminths, and ectoparasites.

*Plasmodium*, a kind of protozoa and the causative pathogen of malaria, causes more deaths globally than all other parasitic diseases. Several aptamers targeting *Plasmodium* have been developed and some have been used for diagnosis. Most *Plasmodium*-specific aptamers have been summarized previously 218, excluding a few novel aptamers with malaria diagnosis applications. To select an aptamer targeting *P. falciparum*, Singh et al. immobilized *P. falciparum* glutamate dehydrogenase (PfGDH, for SELEX) or human glutamate dehydrogenase (hGDH, for counter-SELEX) onto PVDF membranes for protein-SELEX 219. They obtained a 90-mer DNA aptamer with a binding affinity of 0.5 μM. To detect PfGDH in serum samples, they 1) coupled this aptamer with carbon dots as the probe 219, 2) coupled the aptamer with dye as the catcher for an enzyme-catalyzed reaction 220, and 3) applied this aptamer as the capture in a field-effect transistor biosensor 221, resulting in detection limits of 2.85 nM, 63.97 pM, and 48.6 pM, respectively. For rapid diagnostic tests for *Plasmodium* infection, Joseph et al. identified high mobility group box 1 protein (HMGB1) as a biomarker for *Plasmodium* infection using gene expression databases, ribosome profiling analysis, and structural modeling 222. Later, they immobilized biotinylated HMG-box onto streptavidin-coated magnetic beads for protein-SELEX and developed several DNA aptamers of potential use for malaria diagnosis. Minopoli et al. developed an antibody-aptamer plasmonic biosensor to detect *P. falciparum* lactate dehydrogenase (PfLDH) by immobilizing PfLDH-specific antibodies onto gold nanoparticles for PfLDH capture and labeling the PfLDH-specific aptamer to detect the captured PfLDH 223. Using this aptasensor, the detection limit was as low as 1 pg/mL in blood samples. DNA aptamers targeting the surface of erythrocytes infected with *P. falciparum* were developed using cell-SELEX as well 224, 225.

*Trichomonas vaginalis*, an anaerobic, flagellated protozoan parasite, is the causative agent of trichomoniasis. Espiritu and colleagues selected a DNA aptamer against the *T. vaginalis* adhesion protein AP65 using SELEX and developed an enzyme-linked aptamer assay to detect *T. vaginalis* with a detection limit of 8300 cells/mL 226.

*Leishmania infantum* is the causative agent of infantile visceral leishmaniasis. Aptamers against *L. infantum* were developed by different groups and summarized 218. Frezza et al. isolated two DNA aptamers previously identified in an ssDNA library against *L. infantum* H3 (*Li*H3) protein. They labeled either of the two aptamers with digoxigenin for *Li*H3 binding and used an HRP-labeled anti-digoxigenin antibody for detection. Their results indicated that these two DNA aptamers are promising biosensing molecules for leishmaniasis diagnosis 227.

*Cryptosporidium* is a microscopic parasite that causes the diarrheal disease cryptosporidiosis. To detect *Cryptosporidium parvum* oocysts in fresh foods, Iqbal et al. developed 14 DNA aptamers with high affinities for *C. parvum* oocysts and created an electrochemical aptasensor to detect *C. parvum* oocysts in fresh fruit and, raw lake and river waters samples, with detection limits of 100 and 50 oocysts, respectively 228, 229.

*Toxoplasma gondii* is a protozoan parasite that infects most species of warm-blooded animals, including humans, and causes the disease toxoplasmosis. Diagnosis of toxoplasmosis is typically made by serologic testing, namely immunoglobulin G (IgG) detection. Luo et al. obtained two DNA aptamers targeting anti-toxoplasma IgG by SELEX and developed a QD-based aptasensor for anti-toxoplasma IgG detection in serum samples, with a detection limit of 0.1 IU 230. Vargas-Montes et al. developed two DNA aptamers against *T. gondii* rhoptry protein 18, a major virulence factor, from the N40 ssDNA library using protein-SELEX. Based on these two aptamers, they established an ELONA assay for ROP18 protein detection in serum samples, with a limit of 1.56 μg/mL 231. Shen et al. used protein-SELEX to develop DNA aptamers against the *T. gondii* surface antigen protein, a key marker for laboratory diagnosis, from the ssDNA microsphere library modified with indole-dU (w), phenol-dU (Y), and amine-dU(X). Furthermore, they used one of the identified aptamers as the probe in an ELONA assay to detect the native surface antigen protein secreted by *T. gondii* in mouse and human serum samples, with a detection limit of 1.56 μg/mL 232.

## Potential for aptamer technology in communicable disease therapeutics

Though antibodies have achieved marked clinical successes, the side effects caused by their biological activity should not be overlooked 233. In some cases, ideal aptamers may successfully replace antibodies. The therapeutic application of aptamers is also superior to that of antibodies because aptamers are affordable, non-immunogenic, easy to modify, and tissue-permeable 234, 235. Like antibodies, aptamers can bind to and directly affect the function of their targets by binding to the catalytic center or inducing conformational changes in protein structure due to charge change. For example, a 25-mer stem-loop containing DNA aptamer against DNA methyltransferase 1 (DNMT1) can mimic as the DNMT1 substrate to inhibit the enzymatic activity of DNMT1 236. Additionally, a 10-mer DNA aptamer inhibits the activity of bacterial metallo-β-lactamase by inducing conformational change 237. Thus, critical pathogen enzymes and functional proteins can be used as targets for aptamer development to control pathogen propagation. Aptamers can act as activating ligands for enzymes and cell signal pathways. For instance, 2'-5'-oligoadenylate synthetase-targeted RNA aptamers can activate 2'-5'-oligoadenylate synthetase 238. T cell costimulatory receptor (4-1BB)-targeted multivalent RNA aptamers can costimulate activated T cells to promote survival and expansion 239. These immune activators and anti-infection enzymes can be used as targets for aptamer development to improve host immune responses against infection. For functional aptamers, idea targets are protein functional domains. A strategy for functional aptamer selection was developed by Ruff et al. who used a denoted R^*^Y^*^ library for SELEX 240. The library sequence is (R^*^Y^*^)_4_N_4_(R^*^Y^*^)_5_N_3_(R^*^Y^*^)_5_N_4_(R^*^Y^*^)_5_N_3_(R^*^Y^*^)_4_, where R^*^ is 45:5:45:5 A/C/G/T and Y^*^ is 5:45:5:45 A/C/G/T. Relative to the normal N_60_ library, the denoted R^*^Y^*^ library holds a higher degree of secondary structure and can accommodate loops and bulges which are typically found in functional aptamers.

Non-functional aptamers may be applied as a drug delivery vehicle for targeted therapy in infectious disease control. These aptamers can be applied directly or combined with various nanoparticles to deliver siRNA or anti-infection drugs to the infected cells or organs. Below, we list examples of aptamer-mediated infection disease therapeutics to illustrate the virous potential application of aptamers.

### Control of pathogens

**Figure 6:**
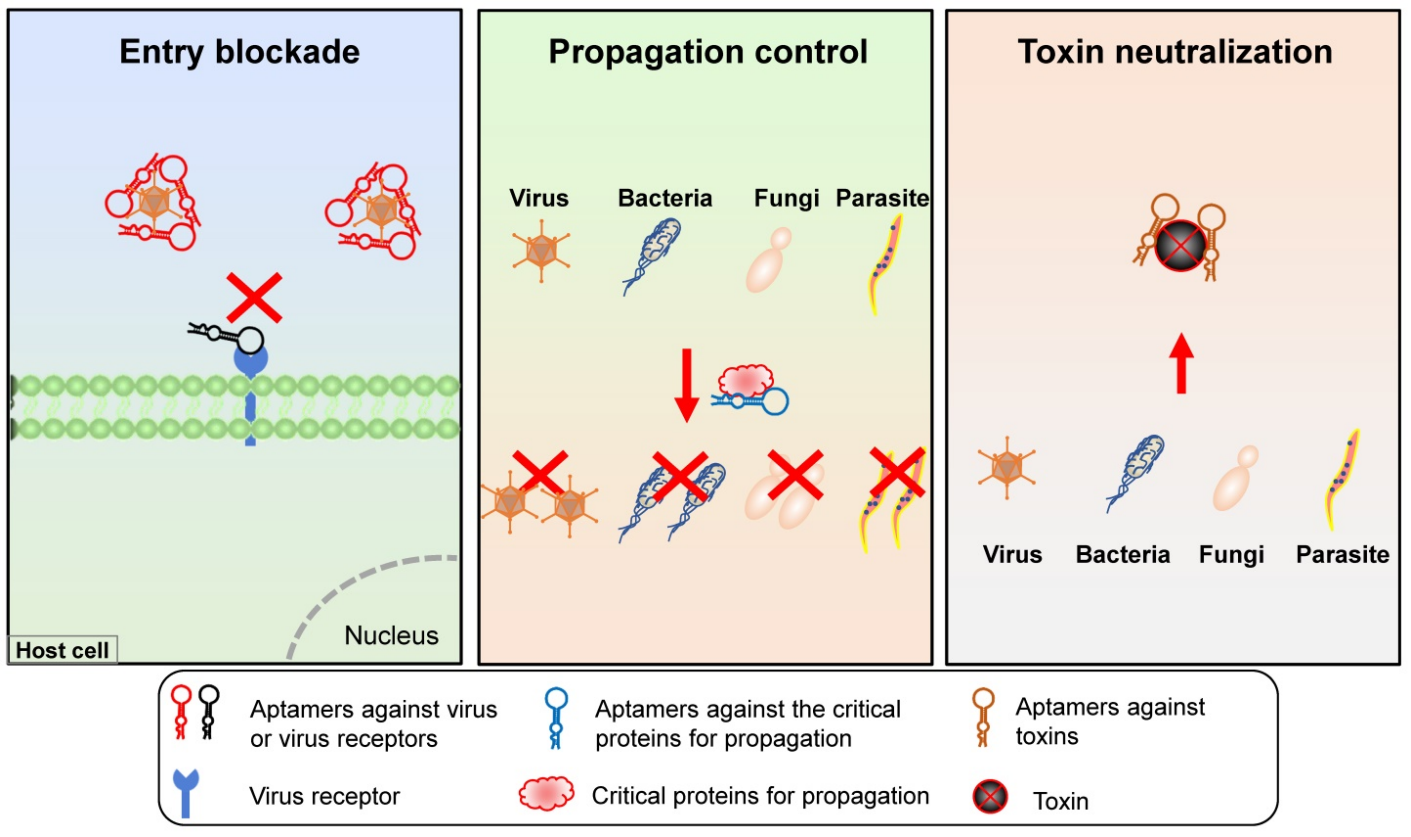
Schematic representation of aptamer applications in pathogen infection control. Three aptamer-based approaches for pathogen infection control include virus entry blockade, pathogen propagation control, and toxin neutralization.

Three types of aptamer technology can be applied for pathogen propagation control (Figure 6): (1) aptamers targeting host or viral surface proteins that mediate host cell entry; (2) aptamers targeting or blocking pathogen proteins necessary for propagation; (3) aptamers targeting pathogen-secreted toxins.

#### Aptamers block host entry of pathogens

Beginning in 2003, the James' group began developing a 2'-F-RNA aptamer (B40) targeting the surface glycoprotein (gp120) of HIV-1 and further identified that this aptamer neutralized HIV-1 infectivity by blocking CCR5, an entry receptor of HIV-1, in a relatively CD4-independent manner 241-243. Mufhandu et al. have since shortened this aptamer (UCLA1) and evidenced its broad-spectrum potency in entry inhibition against several HIV-1 subtype C viruses at low nanomolar concentrations 244. Besides, Zhou et al. developed human CCR5-targeted RNA aptamers capable of inhibiting HIV-1 host cell entry via block of the CCR5 245. Perrone et al. applied the nucleolin-targeted aptamer, AS1411, to block HIV-1 attachment with host cells and identified AS1411 as a new, potent, promising, and safe anti-HIV-1 agent 246. These studies provide a potential therapy for HIV-1 infection and offer models that deepen our understanding of the molecular interactions between HIV-1 and host cells. For therapeutic purpose, those aptamers can be applied in combination in future pre-clinic and clinic studies.

Using protein-SELEX, Jeon et al. obtained a 68-mer DNA aptamer targeting hemagglutinin (HA) of IAV. They further applied this aptamer to inhibit the infectivity of IAV and found that this aptamer inhibited viral infection of different influenza strains with a 90%-99% reduction of viral load in the lungs of treated mice. In terms of the mechanism, this aptamer can block the binding of IAV to target cell receptors and thereby prevent viral invasion of host cells 247. Gopinath et al. developed RNA aptamers specifically targeting the HA of IAV strains A/Panama/2007/1999(H3N2) and A/California/07/2009(H1N1) and illustrated that those aptamers could efficiently inhibit HA-mediated membrane fusion or HA-glycan interaction, providing potential therapeutic agents for the control of IAV infection 77, 248. Likewise, Park et al. selected an RNA aptamer targeting the biologically active HA protein of the subtype H5 avian influenza virus and identified that this aptamer showed significant antiviral efficacy by blocking and inhibiting the receptor-binding domain of viral HA 249. Kwon et al. also developed an RNA aptamer targeting the glycosylated receptor-binding domain of the HA protein and demonstrated its inhibitory effect on virus infection by neutralizing the receptor-binding site of influenza virus HA 250.

**Figure 7:**
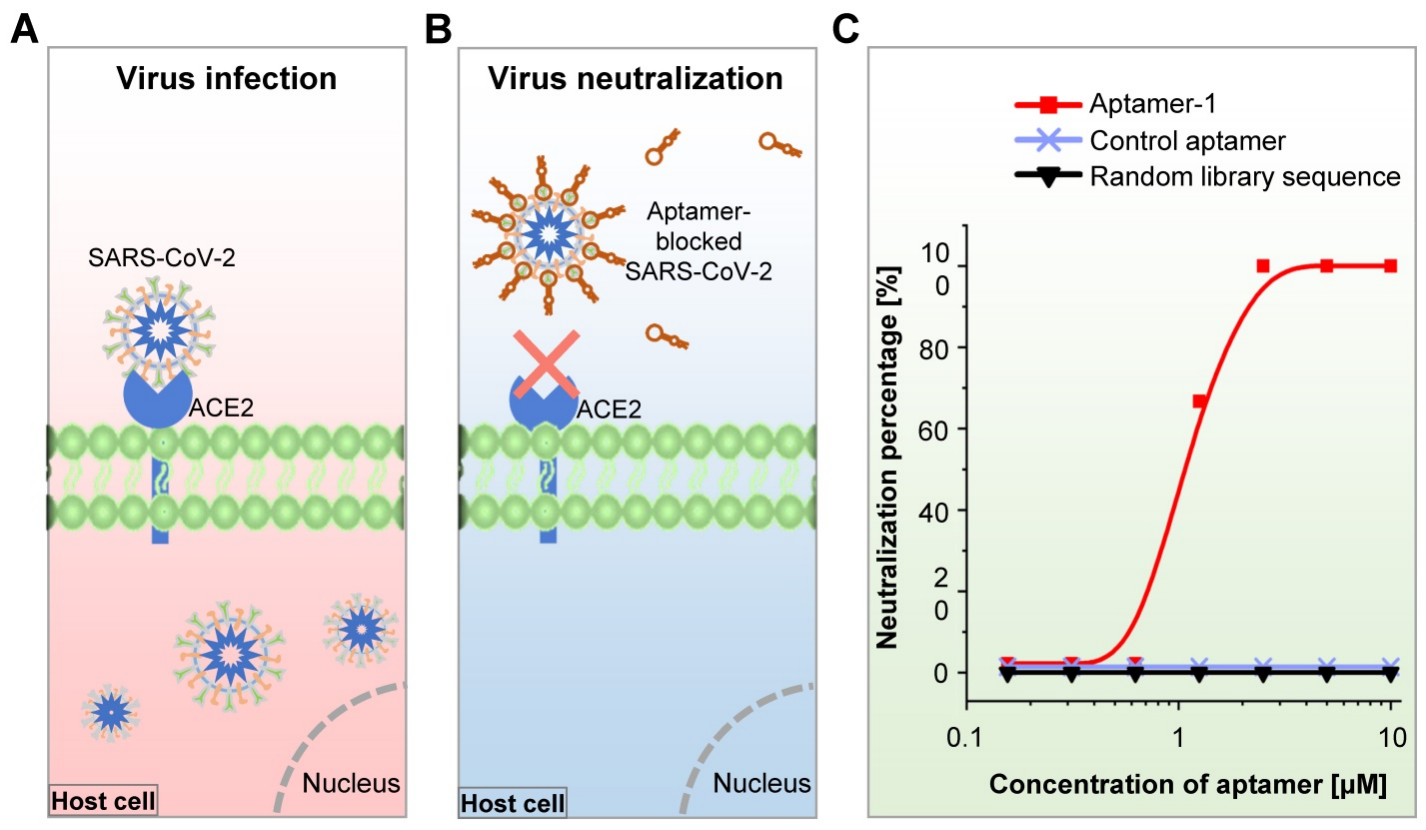
Assays for SARS-CoV-2 virus-host entry blockade. (A) Schematic representation of untreated human ACE2-expressing cells infected by the SARS-CoV-2 virus. (B) Schematic representation of aptamer-treated human ACE2-expressing cells that cannot be infected by the SARS-CoV-2 virus. (C) SARS-CoV-2 virus neutralization efficacy of the RBD-bound aptamer in human ACE2-expressing cells.

During the COVID-19 pandemic, Cleri et al. developed two 51-mer and 67-mer DNA aptamers targeting the RBD of the S1 protein in the SARS-CoV-2 virus and found that these aptamers reduced SARS-CoV-2 infectivity by blocking the interaction between S1-RBD and host cell angiotensin-converting enzyme 2 (ACE2) receptors 251. Sun et al. recently developed an RBD-specific DNA aptamer (cb-CoV2-6C3) with a high binding affinity (K_d_ = 0.13 nM) using RBD-expressed cell-SELEX and found that this aptamer was stable in serum and at ambient temperatures. They further showed that this aptamer can block authentic SARS-CoV-2 virus 252. In the meantime, Mayer et al. developed S protein- or RBD-targeted DNA aptamers for the blockage of SARS-CoV-2 cell entry using protein-SELEX 253. They finally identified one S protein-targeted aptamer that could inhibit the SARS-CoV-2 pseudo-virus cell entry through an RBD-ACE2 interaction-independent way, though this aptamer held a strong binding affinity to S protein (K_d_ = 13.9 nM). We have developed an RBD-specific DNA aptamer using RBD protein-SELEX with a strong binding affinity (K_d_ = 6.05 nM) and found that the aptamer neutralized both pseudo- and authentic SARS-CoV-2 virus and efficiently prevented human host cell infection *in vitro* (Figure 7) 67. Most recently, Yang et al. developed six DNA aptamers using capillary electrophoresis-based SELEX 254. They further testified that one of the aptamers (nCoV-S1-Apt1) could block SARS-CoV-2 spike-pseudovirus infection *in vitro*. In addition to the neutralization antibodies, aptamers are alternative tools that may help control the COVID-19 pandemic.

An RNA and a DNA aptamer targeting gD, a critical protein for herpes simplex virus 1 (HSV-1) entry, were found to interfere with the interaction between gD protein and HSV-1 target cell receptor, indicating that these aptamers can be applied as chemical agents to control HSV-1 infection 255, 256. Yang et al. developed DNA aptamers against the envelope protein of HCV and found that this aptamer inhibited virus binding to host cells, showing an antiviral efficacy 257. To block DENV2 virus-host cell entry, Gandham et al. developed a thioaptamer binding adjacent to a known neutralizing antibody binding site of the envelope protein domain III 258, providing a feasible strategy for the selection of antiviral aptamers.

In the context of animal welfare and agriculture progress, several aptamers have been developed to control viral diseases. To block host entry by Bovine herpesvirus 1 (BoHV-1), Xu et al. used SELEX to develop a DNA aptamer against the BoHV-1 virus that efficiently inhibited viral entry of BoHV-1 in bovine kidney cells, indicating that this aptamer is a potential therapeutic agent for control of BoHV-1 infection in cattle 259. DNA aptamers targeting the S10 protein of grass carp reovirus were also developed and further applied to block viral entry, providing a potential treatment tool for viral diseases in fish 260.

Interestingly, aptamers not only block viral host entry but also block the entry of some bacteria. Kalra et al. developed G-quadruplex-forming DNA aptamers against HupB protein in *M. tuberculosis* and found these aptamers could bind to surface-located HupB to block *M. tuberculosis* entry into THP-1 monocytic cells 261.

#### Aptamers inhibit pathogen propagation

Aptamers can impair the function of target molecules by directly inactivating or blocking them to inhibit their subsequent functions. Early in 1995, Rando et al. found that a guanosine and thymidine-composed oligonucleotide (I100-15) could reduce viral-specific transcripts of the HIV-1 virus 262. Subsequently, the same team modified I100-15 with a hydroxyl moiety at its 3' terminus and a single phosphorothioate internucleoside linkage at both the 5' and 3' ends and named the new aptamer T30177. They found that T30177 inhibited the HIV-1 integrase enzyme to suppress the production of HIV-1 263. Later, Virgilio et al. optimized this aptamer by replacing the ninth thymidine with 5-hydroxymethyl-2′-deoxyuridine residues. The optimized aptamer more strongly inhibited the HIV-1 integrase enzyme 264. Another target for HIV-1 propagation control is reverse transcriptase (Rev). DNA and RNA aptamers targeting HIV-1 Rev were developed and found to inhibit HIV replication *in vitro* and *in vivo* 84, 265-268. The inhibitory function of these aptamers resulted from their ability to interfere with Rev binding to viral primers/templates. Recently, the binding interface for the RNA aptamer targeting HIV-1 Rev was extensively analyzed, illuminating features at the Rev-aptamer interface responsible for recognition specificity that may facilitate the development of aptamers against Rev for HIV-1 replication inhibition 269. In addition to Rev, other HIV-1 proteins, including Tat protein 81, Gag polyprotein 270, and aspartyl protease 271 were also investigated as targets for aptamer development to inhibit HIV-1 propagation.

Other aptamer-based viral replication inhibition studies were performed on HCV and other viruses. RNA aptamers consisting of 2'-hydroxyl- or 2'-fluoropyrimidines against NS5B replicase of HCV were developed and used to inhibit HCV replication, as these aptamers can act as potent decoys to competitively impede replicase-catalyzed RNA synthesis activity 272. Another research team developed HCV NS2-, core protein-, or NS5A-targeted DNA aptamers to inhibit HCV replication. These groups demonstrated that the antiviral effects of these aptamers result from: (1) aptamer binding to the N-terminal of NS2 to sterically hinder the interaction of NS2 with NS5A protein 273, (2) aptamer binding to a core protein to repress the virion production by disrupting the localization of the core with lipid droplets and to inhibit the assembly of infectious viral particles by disrupting the association between core protein and viral RNA 90, and (3) aptamer binding to NS5A to prevent the interaction of NS5A with the core protein 274. Orabi et al. developed a DNA aptamer targeting the matrix binding domain of the HBV capsid and found that this aptamer had an inhibitory efficacy of 47% on HBV virion production 275. Lee and colleagues developed RNA aptamers modified with 2'-O-methyl pyrimidines or 2'-fluorine against the methyltransferase of Japanese encephalitis virus or DENV2 and revealed that these aptamers efficiently inhibited the activity of viral methyltransferases and interfered with virus production in cells 107, 276. Oligonucleotide aptamers for replication inhibition of human papillomavirus type 16, bovine herpesvirus-1, and RGNNV were developed and characterized 277-280. Notably, to control the SARS-CoV virus, Jang et al. developed an RNA aptamer pool containing an AG-rich conserved sequence of 10-11 nucleotides against the NTPase/helicase of SARS-CoV and found that this aptamer pool efficiently inhibited the double-stranded DNA unwinding activity of the helicase by up to ~85% with an IC_50_ value of 1.2 nM 95. Future studies should investigate whether this aptamer pool can inhibit the activity of the helicase of SARS-CoV-2.

Nucleotide aptamers can be applied to inhibit the replication of viruses, bacteria, fungi, and parasites. A DNA aptamer targeting polyphosphate kinase 2, a critical protein of *M. tuberculosis*, inhibited the enzyme activity of its target to weaken the metabolism of *M. tuberculosis* and reduce bacterial persistence and virulence 281. Bachtiar et al. developed RNA aptamers against yeast cells and identified one that inhibited biofilm and hyphal formation in *C. albicans* 282. Vahed et al. found that the G-rich VEGF aptamer was a potential inhibitor for the chitin biosynthesis pathway of *Saccharomyces cerevisiae*, an opportunistic yeast that causes health issues 283. An RNA aptamer containing a “GUUG” motif and interacting with the polyadenylation factor EhCFIm25 of *Entamoeba histolytica* impaired EhCFIm25 function, thereby inhibiting parasite proliferation and inducing *E. histolytica* cell death 284.

#### Aptamers neutralize microbial toxins

Microbial toxins exacerbate infection and disease by directly damaging host tissues and disabling the immune system. Some microbial toxins are stable in food over long periods and cause food poisoning disease. Aptamers binding to either microbial toxins or the receptors of microbial toxins can block or reduce toxic effects. An RNA aptamer targeting the light chain of type A botulinum neurotoxin of *Clostridia botulinum* was developed and successfully inhibited endopeptidase activity, with IC_50_ in a low range 285. To neutralize the α-toxin of *S. aureus*, Vivekananda et al. developed DNA aptamers using SELEX and identified four aptamers that significantly inhibited α-toxin-mediated cell death in Jurkat T cells 286. Wang et al. developed neutralizing DNA aptamers against staphylococcal enterotoxins that inhibited enterotoxin-mediated proliferation and cytokine secretion in human peripheral blood mononuclear cells 287, 288. Interestingly, El-Aziz et al. developed DNA aptamers against αC-conotoxin PrXA, a marine snail ultrafast-killing paralytic, and employed those aptamers and αC-conotoxin PrXA in a model to study the utility of aptamers for bioterrorism incidents. They concluded that DNA aptamers are fast-acting, safe, and cheap antidotes to lethal toxins at risk of misuse in bioterrorism and offered an alternative method to treat cases of envenomation toxin other than donor sera 289. Lahousse et al. developed DNA aptamers for anthrax lethal factor detection. One of these aptamers inhibited anthrax lethal factor protease activity against mitogen-activated protein kinases, providing a novel inhibitor of anthrax lethal factor 290. Aptamers should be further tested for their potential neutralizing functions for microbial toxins.

### Targeted therapy

Because aptamers specifically bind to their targets, they can be applied as vehicles for anti-cancer or anti-pathogen agents to direct drugs to specific locations 291. RNA interference therapy for HIV infection is a safe, effective, and durable approach for HIV/AIDS treatment 292. Akkina and Rossi used the HIV gp120-targeted 2′-fluoro-modified RNA aptamer for siRNA agent delivery to inhibit the expression of gp160 or Tat/Rev of HIV and prevent virus-induced helper CD4+ T cell decline 293-295. Further, they improved the aptamer-guided siRNA delivery system by chemically synthesizing the aptamer with a 3′ 7-carbon linker, which in turn was attached to a 16-nucleotide 2′ OMe/2′ Fl GC-rich bridge sequence 296. With this design, multiple siRNAs can noncovalently bind and interchange a single aptamer for concurrent delivery. Subsequently, they applied this improved aptamer-vehicle for co-delivery of HIV Tat/Rev, CD4, and TNPO3 siRNAs or delivery of small noncoding RNAs to inhibit HIV-1 infection 296, 297. The RNA aptamer against CD4 was applied as the vehicle for either HIV gag/vif siRNA or host CCR5 siRNA delivery to inhibit HIV infection 298. In addition to AIDS, aptamer-guided siRNA delivery was also applied to control animal viral diseases. Zhou et al. developed DNA aptamers against RGNNV-infected grouper brain cells using cell-SELEX and identified one aptamer that could be applied for targeted siRNA delivery to inhibit viral infection 299. These studies provide promising targeted gene-therapies for viral infection, which warrant further investigation in the context of other infectious viruses.

Aptamer-based target delivery has also been applied to control bacterial and parasitic diseases. A DNA aptamer targeting *S. aureus* was used as the guidance and drug-release gate of a vancomycin-loaded nano-capsule or the teicoplanin-encapsulated nanoparticle of poly lactic-co-glycolic acid to inhibit *S. aureus* propagation 300, 301. To improve the antibacterial efficiency of TiO_2_, Song et al. conjugated an aptamer cocktail containing three DNA aptamers against *E. coli* with the TiO_2_ particle 302. Relative to non-aptamer- and single aptamer-conjugated TiO_2_ particles, the aptamer cocktail-conjugated TiO_2_ particle more efficiently inactivated *E. coli*. This study provides an improvement strategy for targeted bacterial control. A DNA aptamer against *V. vulnificus* was exploited as the navigator for the antimicrobial peptide (HPA3P^His^)-loaded GNP to successfully control *V. vulnificus* infection *in vitro* and *in vivo* 303. Likewise, synthesized DNA aptamer and silver nanocluster conjugates enhanced antibacterial efficiency and reduced host-toxic effects *in vitro* and* in vivo* 304. Aptamer-based therapeutics for parasitic diseases have also been explored. Homann and Goringer demonstrated that RNA aptamers can be used as 'piggy-back' molecules to deliver compounds/toxins to the lysosomal compartment of African trypanosomes 305. Cardenas-Guerra et al. delivered antisense DNA into *Trypanosoma cruzi* using virus-like protein-based nanoparticles to induce cell damage and death 306, indicating that aptamer-mediated guidance of antisense DNA or other drugs may be an additional promising therapeutic strategy to control *T. cruzi*-induced disease.

Because pathogen-targeted aptamers can be easily obtained either from published reports or via various SELEX procedures, aptamer-guided delivery of anti-pathogenic agents is a promising therapy for infectious diseases that is efficient, specific, and carries a low risk of broad-spectrum drug resistance.

### Immune system improvement

**Figure 8:**
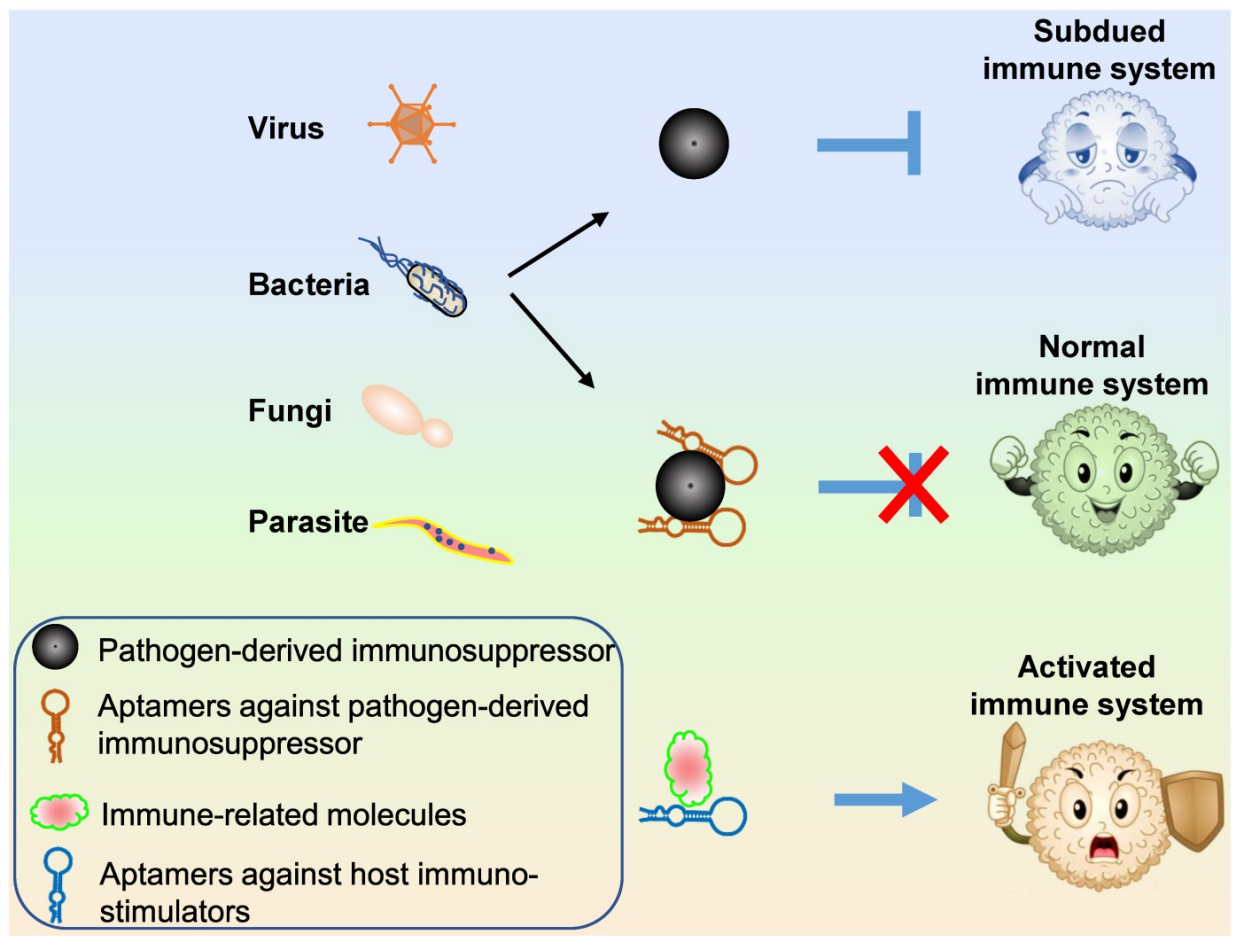
Schematic representation of aptamer applications in host immunity improvement during pathogen infection. Aptamers can specifically bind and block pathogen-derived immunosuppressors to maintain normal host immune system function. Alternatively, aptamers may activate host immunostimulators to boost host immunity again pathogens. Cartoon portraits representing the immune system are derived from Google images created by Lorelyn Medina.

The primary function of the host immune system is protection from viral, bacterial, and parasitic invasion. Host immune responses are initiated by recognition of pathogenic antigens during the invasion. The recognized signal is transduced and amplified by various immune signaling transduction pathways. To defend against these responses, pathogens use specific strategies to suppress the host immune system (Figure 8). One of these strategies is the production of immunosuppressors either via secretion or presentation at the host or autologous cell surface 307. In this context, aptamers can be applied to block pathogenic immunosuppressors to recover the host immune system (Figure 8). The major function of the NS1 of influenza virus is to inhibit innate immune responses by suppressing the induction of interferon (IFN). To block NS1 activity, Woo et al. developed DNA and RNA aptamers against NS1 protein using SELEX and revealed that those aptamers increased the production of IFN-β to inhibit viral replication by blocking NS1 activity 308, 309. Mannose-capped lipoarabinomannan (Man-LAM) is a surface lipoglycan of *M. tuberculosis* that suppresses host immune responses. Pan et al. developed a DNA aptamer targeting Man-LAM using SELEX and found that this aptamer significantly inhibited Man-LAM-induced immunosuppression of CD11c^+^ dendritic cells (DCs) and enhanced the *M. tuberculosis* antigen-presenting activity of DCs for naïve CD4+ Th1 cell activation 310. Later, the same group used SELEX to develop another DNA aptamer against Man-LAM of *M. bovis* and further identified that this new aptamer blocked the binding of Man-LAM with its receptor and induced Man-LAM-CD44 signaling to enhance M1 macrophage and Th1 cell activation *in vitro* and *in vivo* 311. Another example can be found in control the infection of the Ebla virus by aptamers. In Ebola-infected cells, the binding of host karyopherin alpha 1 by Ebola virus VP24 protein prevents the downstream signal to inhibit host immune responses. Tanaka et al. developed RNA aptamers against the VP24 protein through capillary electrophoresis SELEX and further identified the resultant aptamers could block the binding of VP24 to karyopherin alpha 1 312. Mechanistically, most pathogens are capable of suppressing host immune system attacks. Aptamer blockade of pathogenic immunosuppressors is a promising therapy to recover host immune system functions and restore defense mechanisms.

Aptamers can also strengthen host immune responses by either activating or inhibiting key immune and immune-regulating molecules, respectively (Figure 8). The phosphorylated DNA aptamer against toll-like receptor 9 (TLR9) is an intracellular TLR9 agonist that activates TLR signaling pathways to elicit local or adaptive immune responses against bacterial infection 313. A CD44-targeting DNA aptamer conjugated with discoidal silicon mesoporous microparticles were used to control *M. tuberculosis* infection by inducing *M. tuberculosis*-infected macrophage death and activating cellular immunity 314. Hwang et al. obtained RNA aptamers that specifically target RIG-I protein, an intracellular pathogen pattern recognition receptor, and verified that one of these aptamers blocks the replication of NAV, vesicular stomatitis virus, and influenza virus in infected host cells by activating the RIG-I signaling pathway and subsequently inducing IFNα/β 315. Interestingly, Avci-Adali et al. incubated synthetic ssDNA aptamers with fresh human blood at 37˚C for two or four hours to measure the immune activation potential of synthetic aptamers 316. Using transcriptome sequencing, they found that those DNA aptamers preferred to induce immune and inflammatory responses through TLR signaling pathways, indicating that DNA aptamers are potential immune activators. In addition, a TIM-3 targeted RNA aptamer was developed and found to block the interaction of TIM-3 with Galectin-9 to enhance T cell functions 317. More aptamers against immune-related molecules have been developed and reviewed previously 318-320. Future investigations assessing the utility of these aptamers as antibiotic-independent therapeutic agents for infectious disease control are warranted.

## Conclusions and perspectives

Oligonucleotide aptamers can be developed and identified rapidly using high-throughput sequencing technology and various SELEX strategies. For pathogen detection or disease diagnosis, aptamers against whole pathogens, pathogen components, pathogen or disease markers, microbial toxins, or pathogen-infected host cells can be developed. These aptamers can then be combined with various platforms to optimize detection speed, convenience, cost-effectiveness, and simplicity. For therapeutic purposes, aptamers can be selected against (1) surface components of pathogens or host cell receptors to block host cell entry or deliver drugs, (2) indispensable proteins and enzymes to hamper pathogen propagation, (3) microbial toxins to relieve symptoms caused by toxins, or (4) pathogenic immunosuppressors or host immune-related molecules to augment host immune system function. Since aptamers against the same pathogen were broadly developed, it is worth trying to prepare aptamer cocktails for infectious disease control.

Though oligonucleotide aptamers are well suited for biomedical applications and promising tools for disease diagnosis and therapy, there are drawbacks in the development and application processes that impede commercial translation. First, the cost of production. Although aptamers are cheap, those aptamer modifications, aptamer-immobilizing matrix, and detection devices are costly, resulting in the overall cost is not significantly cheaper than existing antibody-based detection methods. Therefore, researchers should pay more attention to those precise instrument-independent aptamer-based detection methods. Second, optimal environmental binding conditions for certain aptamers are unalterable. Generally, aptamer-SELEX is performed in either one or a few kinds of binding buffers, such as PBS-based, cell culture medium-based, or Tris-buffered saline-based. This variability leads to non-specific binding and reduced binding affinity of selected aptamers in different solutions, potentially resulting in non-repeatable aptamer binding and limiting aptamer applicability in pathogen detection and disease diagnosis. Thus, during aptamer selection for disease diagnosis and treatment purposes, aptamer binding abilities in different solutions, including human plasma, should be considered. Alternatively, aptamers can be chemically modified to improve their stabilities and binding affinities in different solutions 321. Third, because oligonucleotide aptamers have a negative charge, they tend to attach positively charged objects, producing aptamers that are non-specific. Optimized strategies can be employed during SELEX procedures to avoid this problem 322. For instance, adding polyanionic detergents, surfactants, or cationic chelators to the binding buffer during SELEX can mask non-specific electrostatic interactions. Fourth, the actual binding affinity and specificity of some of the aptamers maybe not as high as they reported, which limits the application to the experimental research stage. Fifth, the degree of market recognition of aptamer is low. Few companies are willing to pay for the commercialization of aptamer-related patents. Herein, we suggest researchers aim to develop appliable products rather than publishing papers, as such papers do not substantively promote the progress of aptamer application in the clinic.

As the development of aptamer applications is in its early stages, more and more commercial products should be exploited. In this way, the degree of aptamer market recognition can be increased. To implement this plan, studies focusing on the theoretical basis of aptamer binding should be performed. On an explicitly theoretical basis, one could design aptamers targeting objects of interest *in silico* and select functional aptamers. As the theoretical basis was established based on data related to actual aptamer binding cases, it is premature to conclude that aptamer binding characteristics were inconstant. More reliable data regarding the chemical, physical, and structural mechanisms of aptamer binding should be collected to consummate the theoretical basis in the future.

In summary, numerous aptamers have been developed against various pathogens. The sequence information, binding affinity, developmental conditions for SELEX, and detection limits of these aptamers are summarized herein. Most of the described aptamers were studied with the intent to detect pathogens or treat infectious diseases. Currently available data indicate that aptamer-based diagnosis and treatment platforms for communicable diseases are both feasible and worth future investigation and development. Although many unknown theories and factors limit aptamer development at the current phase of experimental research, we expect that aptamer products suitable for the diagnosis and treatment of communicable diseases will be identified as technology advances. Importantly, to translate aptamer-based studies into a technology with clinical application, multidisciplinary research, cross talk, and collaboration will be indispensable. Through synergetic and collaborative research that includes chemists, microbiologists, clinicians, and industry, aptamer-based diagnostics and therapeutics for infectious diseases may soon become available clinically.

## Supplementary Material

Supplementary tables.

## Figures and Tables

**Table 1:** Partially representative oligonucleotide aptamers for virus detection

Virus	Aptamer target	Kd	Detection method	Detection limit	Reference
Duck parvovirus	Whole virus	467 nM	AuNP-based colorimetric assay	1.5 EID_50_	55
Ebola virus	GP protein	4.1 nM	Magnetism-controlled detection chip	4.2 ng/mL	56
Foot and mouth disease virus	VP1 structural polypeptide	-	Competitive FRET	25 ng/mL	57
Hepatitis B virus	E antigen	0.4 nM	ELONA	0.1 ng/mL	58
Hepatitis C virus	Core antigen	100 nM	ALFA	10 pg/mL	59
HIV-1	Reverse transcriptase	0.71 nM	Aptamer-based radioactivity assay	100 virions	60
Influenza virus H1N1	whole virus	55.14 nM	Aptamer-trapping PCR or fluorescence probe	6.4×10^-3^ HAU or 0.032 HAU	61, 62
Influenza virus H5N1	HA1 proteins	15.3 nM	Electrochemical aptasensor or surface-enhanced Raman scattering aptasensor	100 fM or 10^-4^ HAU	63, 64
SARS-CoV	N protein	1.65 nM	Nanoarray aptamer chip or fluorescent aptamer probe	2 pg/mL or 0.1 pg/mL	65, 66
SARS-CoV-2	RBD	6.05 nM	Aptamer-qPCR signal amplification	8 pM	67
Severe fever with thrombocytopenia syndrome virus	Nucleocapsid protein	0.8 μM	ELONA	9 pg/mL	68
Zika virus	NS1 protein	45 pM /134 nM	ELONA	100 ng/mL	69
Newcastle disease virus	Whole virus	31 nM /78.1 nM	ELONA	1.2 EID_50_/mL	70

“-” indicates missing information

**Table 2:** Partially representative oligonucleotide aptamers for the detection of bacteria

Virus	Aptamer target	Kd	Detection method	Detection limit	Reference
*B. bifidum*	Whole cell	10.69 nM	Colorimetric bioassay	10000 CFU/mL	121
*C. jejuni*	Whole cell	292.8 nM	Colorimetric aptasensor	100 CFU/mL	122
*E. coli 8739*	Outer membrane proteins	1.65 nM	Competitive FRET	30/mL	123
*E.coli* O157:H7	Whole cell	10.2 nM	QCM aptasensor	1460 CFU/mL	124
*L. monocytogenes*	Whole cell	74.4 or 48.74 nM	Antibody-trapping PCR or fluorescence probe	5 or 75 CFU/mL	125, 126
*M. tuberculosis*	Ag85A/HspX antigen	63 nM or 90 nM	Fluorescence probe or ENOLA	1.5 nM or 10 pg	127-130
*N. meningitidis*	Whole cell	28.3 nM	Flow cytometry	100 CFU/mL	131
*P. mirabilis*	Whole cell	4.1 nM	Fluorescent aptasensor	526 CFU/mL	132
*P. aeruginosa*	Whole cell	17.27 nM	Fluorometric aptamer probe	1 CFU/mL	133
*S. enterica*	Vi-antigen	638.6 nM	Electrochemical aptasensor	100 pg/mL	134
*S. typhimurium*	Whole cell	58.5 nM	Electrochemical impedance aptasensor	80 CFU/mL	135
*S. enteritidis*	Whole cell	309 nM	Aptamer-based colorimetric assay	1000 CFU/mL	136
*S. dysenteriae*	Whole cell	23.47 nM	Impedimetric aptasensor	1 CFU/mL	137
*S. aureus*	Whole cell	1.39 μM	Electrochemical aptasensor	1 CFU/mL	138, 139
*V. fischeri*	Whole cell	128 pM /1.25 nM	ALFA	40 CFU/mL	140
*V. parahemolyticus*	Whole cell	16.88 nM	Aptamer-based trapping/Colorimetric aptasensor	10 CFU/mL	51, 141, 142
*V. vulnificus*	Whole cell	26.8 nM	Aptamer-trapping PCR	8 CFU/mL	143

## Abbreviation

ACE2: angiotensin-converting enzyme 2
ALFA: aptamer-based lateral flow assay
ASPV: apple stem pitting virus
BoHV-1: Bovine herpesvirus 1
CP: colored or fluorescent particle
DENV: dengue virus
ELISA: enzyme-linked immunosorbent assay
ELONA: enzyme-linked oligonucleotide assay
FRET: fluorescence resonance energy transfer
GDH: glutamate dehydrogenase
GNP: gold nanoparticle
GO: graphene oxide
HA: Hemagglutinin
HAU: hemagglutination unit
HBV/HCV: hepatitis B/C virus
HIV: human immunodeficiency virus
IAV/IBV: influenza A/B virus
IFN: interferon
LDH: lactate dehydrogenase
LPS: lipopolysaccharide
Man-LAM: mannose-capped lipoarabinomannan
MDPV: Muscovy duck parvovirus
NAV: Newcastle avian virus
NP: nucleocapsid protein
NS1: non-structural protein 1
QCM: quartz crystal microbalance
QD: quantum dot
RBD: receptor-binding domain
Rev: reverse transcriptase
qPCR: real-time quantitative PCR
SARS: severe acute respiratory syndrome
SELEX: systematic evolution of ligands by exponential enrichment
SFTS: severe fever with thrombocytopenia syndrome
SPR: surface plasmon resonance
ssDNA/ssRNA: single-stranded DNA/RNA
WHO: World Health Organization

## References

[1] Davydova A, Vorobjeva M, Pyshnyi D, Altman S, Vlassov V, Venyaminova A (2016). Aptamers against pathogenic microorganisms. Crit Rev Microbiol.

[2] Li HY, Jia WN, Li XY, Zhang L, Liu C, Wu J (2020). Advances in detection of infectious agents by aptamer-based technologies. Emerg Microbes Infect.

[3] Banerjee S, Nilsen-Hamilton M, Aptamers for infectious disease diagnosis [Online first] (2019). In E. Coli infections - importance of early diagnosis and efficient treatment, Rodrigo, L, Ed. IntechOpen.

[4] Zhang T, Lu Y, Deng S, Deng R (2021). Aptamers for the diagnosis of infectious diseases. In Dong, Y, Ed. Aptamers for medical applications: From diagnosis to therapeutics. Singapore: Springer Singapore.

[5] Nimjee SM, White RR, Becker RC, Sullenger BA (2017). Aptamers as therapeutics. Annu Rev Pharmacol Toxicol.

[6] Buglak AA, Samokhvalov AV, Zherdev AV, Dzantiev BB (2020). Methods and applications of in silico aptamer design and modeling. Int J Mol Sci.

[7] Shigdar S, Macdonald J, O'Connor M, Wang T, Xiang D, Al Shamaileh H (2013). Aptamers as theranostic agents: modifications, serum stability and functionalisation. Sensors (Basel).

[8] Dunn MR, Jimenez RM, Chaput JC (2017). Analysis of aptamer discovery and technology. Nat Rev Chem.

[9] Drolet DW, Moon-McDermott L, Romig TS (1996). An enzyme-linked oligonucleotide assay. Nat biotechnol.

[10] Toh SY, Citartan M, Gopinath SC, Tang TH (2015). Aptamers as a replacement for antibodies in enzyme-linked immunosorbent assay. Biosens Bioelectron.

[11] Zhou L, Wang MH, Wang JP, Ye ZZ (2011). Application of biosensor surface immobilization methods for aptamer. Chin J Anal Chem.

[12] Balamurugan S, Obubuafo A, Soper SA, Spivak DA (2008). Surface immobilization methods for aptamer diagnostic applications. Anal Bioanal Chem.

[13] Dong Y (2019). Aptamers for analytical applications: Affinity acquisition and method design. 1 ed. Weinheim, Germany: Wiley-VCH.

[14] Koczula KM, Gallotta A (2016). Lateral flow assays. Essays Biochem.

[15] Wong R, Tse H (2009). Lateral Flow Immunoassay. Humana Press.

[16] Schüling T, Eilers A, Scheper T, Walter J (2018). Aptamer-based lateral flow assays. AIMS Bioeng.

[17] Jauset-Rubio M, El-Shahawi MS, Bashammakh AS, Alyoubi AO, O′Sullivan CK (2017). Advances in aptamers-based lateral flow assays. TrAC, Trends Anal Chem.

[18] Zhu C, Zhao Y, Yan M, Huang Y, Yan J, Bai W (2016). A sandwich dipstick assay for ATP detection based on split aptamer fragments. Anal Bioanal Chem.

[19] Chen A, Yan M, Yang S (2016). Split aptamers and their applications in sandwich aptasensors. TrAC, Trends Anal Chem.

[20] Wang T, Chen L, Chikkanna A, Chen S, Brusius I, Sbuh N (2021). Development of nucleic acid aptamer-based lateral flow assays: A robust platform for cost-effective point-of-care diagnosis. Theranostics.

[21] Hianik T (2018). Aptamer-based biosensors. In Wandelt, K, Ed. Encyclopedia of Interfacial Chemistry. Elsevier.

[22] Damborsky P, Svitel J, Katrlik J (2016). Optical biosensors. Essays Biochem.

[23] Chen J, Fang Z, Liu J, Zeng L (2012). A simple and rapid biosensor for ochratoxin A based on a structure-switching signaling aptamer. Food Control.

[24] Sabet FS, Hosseini M, Khabbaz H, Dadmehr M, Ganjali MR (2017). FRET-based aptamer biosensor for selective and sensitive detection of aflatoxin B1 in peanut and rice. Food Chem.

[25] Jeon W, Lee S, Manjunatha DH, Ban C (2013). A colorimetric aptasensor for the diagnosis of malaria based on cationic polymers and gold nanoparticles. Anal Biochem.

[26] Yang C, Wang Y, Marty JL, Yang X (2011). Aptamer-based colorimetric biosensing of Ochratoxin A using unmodified gold nanoparticles indicator. Biosens Bioelectron.

[27] Sassolas A, Blum LJ, Leca-Bouvier BD (2009). Electrochemical Aptasensors. Electroanalysis.

[28] He L, Huang R, Xiao P, Liu Y, Jin L, Liu H (2021). Current signal amplification strategies in aptamer-based electrochemical biosensor: A review. Chin Chem Lett.

[29] Wang R, Wang L, Callaway ZT, Lu H, Huang TJ, Li Y (2017). A nanowell-based QCM aptasensor for rapid and sensitive detection of avian influenza virus. Sens Actuators, B.

[30] Bayramoglu G, Ozalp VC, Oztekin M, Arica MY (2019). Rapid and label-free detection of Brucella melitensis in milk and milk products using an aptasensor. Talanta.

[31] Wang R, Li Y (2013). Hydrogel based QCM aptasensor for detection of avian influenza virus. Biosens Bioelectron.

[32] Zhang P, Zhao N, Zeng Z, Feng Y, Tung CH, Chang CC (2009). Using an RNA aptamer probe for flow cytometry detection of CD30-expressing lymphoma cells. Lab Invest.

[33] Sun H, Tan W, Zu Y (2016). Aptamers: versatile molecular recognition probes for cancer detection. Analyst.

[34] Sefah K, Shangguan D, Xiong X, O'Donoghue MB, Tan W (2010). Development of DNA aptamers using cell-SELEX. Nat Protoc.

[35] Percze K, Szakacs Z, Scholz E, Andras J, Szeitner Z, Kieboom CH (2017). Aptamers for respiratory syncytial virus detection. Sci Rep.

[36] Wan Q, Liu X, Zeng Z, Chen Z, Liu Y, Zu Y (2020). Aptamer cocktail to detect multiple species of mycoplasma in cell culture. Int J Mol Sci.

[37] Wang RE, Zhang Y, Cai J, Cai W, Gao T (2011). Aptamer-based fluorescent biosensors. Curr Med Chem.

[38] Shen L, Bing T, Liu X, Wang J, Wang L, Zhang N (2018). Flow cytometric bead sandwich assay based on a split aptamer. ACS Appl Mater Interfaces.

[39] Chinnappan R, AlAmer S, Eissa S, Rahamn AA, Abu Salah KM, Zourob M (2017). Fluorometric graphene oxide-based detection of Salmonella enteritis using a truncated DNA aptamer. Microchim Acta.

[40] Liu Y, Mao G, Wang W, Tian S, Ji X, Liu M (2019). In situ synthesis of photoluminescence-quenching nanopaper for rapid and robust detection of pathogens and proteins. Chem Commun (Camb).

[41] Draz MS, Shafiee H (2018). Applications of gold nanoparticles in virus detection. Theranostics.

[42] Fischer C, Klockmann S, Wessels H, Hunniger T, Schrader J, Paschke-Kratzin A (2016). Aptamer-based trapping of phytosphingosine in urine samples. J Biotechnol.

[43] Fischer C, Fischer M (2017). Aptamer-based trapping: Enrichment of bacillus cereus spores for real-time PCR detection. Methods Mol Biol.

[44] Wu S, Duan N, Zhang W, Zhao S, Wang Z (2016). Screening and development of DNA aptamers as capture probes for colorimetric detection of patulin. Anal Biochem.

[45] Hünniger T, Fischer C, Wessels H, Hoffmann A, Paschke-Kratzin A, Haase I (2015). Food sensing: Selection and characterization of DNA aptamers to Alicyclobacillus spores for trapping and detection from orange juice. J Agric Food Chem.

[46] Joshi R, Janagama H, Dwivedi HP, Senthil Kumar TM, Jaykus LA, Schefers J (2009). Selection, characterization, and application of DNA aptamers for the capture and detection of Salmonella enterica serovars. Mol Cell Probes.

[47] Ozalp VC, Bayramoglu G, Kavruk M, Keskin BB, Oktem HA, Arica MY (2014). Pathogen detection by core-shell type aptamer-magnetic preconcentration coupled to real-time PCR. Anal Biochem.

[48] Suh SH, Dwivedi HP, Jaykus LA (2014). Development and evaluation of aptamer magnetic capture assay in conjunction with real-time PCR for detection of Campylobacter jejuni. LWT Food Sci Technol.

[49] Suh SH, Jaykus LA (2013). Nucleic acid aptamers for capture and detection of Listeria spp. J Biotechnol.

[50] Bodulev OL, Sakharov IY (2020). Isothermal nucleic acid amplification techniques and their use in bioanalysis. Biochemistry (Mosc).

[51] Song S, Wang X, Xu K, Xia G, Yang X (2019). Visualized detection of Vibrio parahaemolyticus in food samples using dual-functional aptamers and cut-assisted rolling circle amplification. J Agric Food Chem.

[52] Cai R, Yin F, Chen H, Tian Y, Zhou N (2020). A fluorescent aptasensor for Staphylococcus aureus based on strand displacement amplification and self-assembled DNA hexagonal structure. Microchim Acta.

[53] Gong Q, Wang J, Ahmad KM, Csordas AT, Zhou J, Nie J (2012). Selection strategy to generate aptamer pairs that bind to distinct sites on protein targets. Anal Chem.

[54] World Health Organization (2018). Managing epidemics: Key facts about major deadly diseases. World Health Organization.

[55] Lu T, Ma Q, Yan W, Wang Y, Zhang Y, Zhao L (2018). Selection of an aptamer against Muscovy duck parvovirus for highly sensitive rapid visual detection by label-free aptasensor. Talanta.

[56] Hong SL, Xiang MQ, Tang M, Pang DW, Zhang ZL (2019). Ebola virus aptamers: From highly efficient selection to application on magnetism-controlled chips. Anal Chem.

[57] Bruno JG, Carrillo MP, Phillips T (2008). Development of DNA aptamers to a foot-and-mouth disease peptide for competitive FRET-based detection. J Biomol Tech.

[58] Liu Y, Le C, Tyrrell DL, Le XC, Li XF (2020). Aptamer binding assay for the E antigen of hepatitis B using modified aptamers with G-quadruplex structures. Anal Chem.

[59] Wang C, Zhang L, Shen X (2013). Development of a nucleic acid lateral flow strip for detection of hepatitis C virus (HCV) core antigen. Nucleosides Nucleotides Nucleic Acids.

[60] DeStefano JJ, Alves Ferreira-Bravo I (2018). A highly sensitive aptamer-based HIV reverse transcriptase detection assay. J Virol Methods.

[61] Lai HC, Wang CH, Liou TM, Lee GB (2014). Influenza A virus-specific aptamers screened by using an integrated microfluidic system. Lab Chip.

[62] Tseng YT, Wang CH, Chang CP, Lee GB (2016). Integrated microfluidic system for rapid detection of influenza H1N1 virus using a sandwich-based aptamer assay. Biosens Bioelectron.

[63] Diba FS, Kim S, Lee HJ (2015). Amperometric bioaffinity sensing platform for avian influenza virus proteins with aptamer modified gold nanoparticles on carbon chips. Biosens Bioelectron.

[64] Kukushkin VI, Ivanov NM, Novoseltseva AA, Gambaryan AS, Yaminsky IV, Kopylov AM (2019). Highly sensitive detection of influenza virus with SERS aptasensor. PLoS One.

[65] Ahn DG, Jeon IJ, Kim JD, Song MS, Han SR, Lee SW (2009). RNA aptamer-based sensitive detection of SARS coronavirus nucleocapsid protein. Analyst.

[66] Roh C, Jo SK (2011). Quantitative and sensitive detection of SARS coronavirus nucleocapsid protein using quantum dots-conjugated RNA aptamer on chip. J Chem Technol Biotechnol.

[67] Liu X, Wang YL, Wu J, Qi J, Zeng Z, Wan Q (2021). Neutralizing aptamers block S/RBD-ACE2 interactions and prevent host cell infection. Angew Chem Int Ed.

[68] Yeom G, Kang J, Jang H, Nam HY, Kim MG, Park CJ (2019). Development of DNA aptamers against the nucleocapsid protein of severe fever with thrombocytopenia syndrome virus for diagnostic application: Catalytic signal amplification using replication protein A-conjugated liposomes. Anal Chem.

[69] Lee KH, Zeng H (2017). Aptamer-based ELISA assay for highly specific and sensitive detection of zika NS1 protein. Anal Chem.

[70] Marnissi B, Kamali-Moghaddam M, Ghram A, Hmila I (2020). Generation of ssDNA aptamers as diagnostic tool for Newcastle avian virus. PLoS One.

[71] Wang R, Zhao J, Jiang T, Kwon YM, Lu H, Jiao P (2013). Selection and characterization of DNA aptamers for use in detection of avian influenza virus H5N1. J Virol Methods.

[72] Bai H, Wang R, Hargis B, Lu H, Li Y (2012). A SPR aptasensor for detection of avian influenza virus H5N1. Sensors (Basel).

[73] Kwon J, Lee Y, Lee T, Ahn JH (2020). Aptamer-based field-effect transistor for detection of avian influenza virus in chicken serum. Anal Chem.

[74] Lum J, Wang R, Hargis B, Tung S, Bottje W, Lu H (2015). An impedance aptasensor with microfluidic chips for specific detection of H5N1 avian influenza virus. Sensors (Basel).

[75] Bhardwaj J, Chaudhary N, Kim H, Jang J (2019). Subtyping of influenza A H1N1 virus using a label-free electrochemical biosensor based on the DNA aptamer targeting the stem region of HA protein. Anal Chim Acta.

[76] Narayan C, Kwon J, Kim C, Kim SJ, Jang SK (2020). Virus-based SELEX (viro-SELEX) allows development of aptamers targeting knotty proteins. Analyst.

[77] Gopinath SC, Kumar PK (2013). Aptamers that bind to the hemagglutinin of the recent pandemic influenza virus H1N1 and efficiently inhibit agglutination. Acta Biomater.

[78] Shiratori I, Akitomi J, Boltz DA, Horii K, Furuichi M, Waga I (2014). Selection of DNA aptamers that bind to influenza A viruses with high affinity and broad subtype specificity. Biochem Biophys Res Commun.

[79] Gopinath SC, Sakamaki Y, Kawasaki K, Kumar PK (2006). An efficient RNA aptamer against human influenza B virus hemagglutinin. J Biochem.

[80] Kim SH, Lee J, Lee BH, Song CS, Gu MB (2019). Specific detection of avian influenza H5N2 whole virus particles on lateral flow strips using a pair of sandwich-type aptamers. Biosens Bioelectron.

[81] Yamamoto R, Katahira M, Nishikawa S, Baba T, Taira K, Kumar PKR (2000). A novel RNA motif that binds efficiently and specifically to the Ttat protein of HIV and inhibits the trans-activation by Tat of transcription in vitro and in vivo. Genes to Cells.

[82] Fatin MF, Rahim Ruslinda A, Gopinath SCB, Arshad MKM, Hashim U, Lakshmipriya T (2019). Co-ordinated split aptamer assembly and disassembly on gold nanoparticle for functional detection of HIV-1 tat. Process Biochem.

[83] Caglayan MO, Ustundag Z (2020). Spectrophotometric ellipsometry based Tat-protein RNA-aptasensor for HIV-1 diagnosis. Spectrochim Acta A Mol Biomol Spectrosc.

[84] DeStefano JJ, Nair GR (2008). Novel aptamer inhibitors of human immunodeficiency virus reverse transcriptase. Oligonucleotides.

[85] DeStefano JJ, Cristofaro JV (2006). Selection of primer-template sequences that bind human immunodeficiency virus reverse transcriptase with high affinity. Nucleic Acids Res.

[86] Liu Y, Wang C, Li F, Shen S, Tyrrell DL, Le XC (2012). DNase-mediated single-cycle selection of aptamers for proteins blotted on a membrane. Anal Chem.

[87] Huang R, Xi Z, Deng Y, He N (2016). Fluorescence based aptasensors for the determination of hepatitis B virus e antigen. Sci Rep.

[88] Xi Z, Huang R, Li Z, He N, Wang T, Su E (2015). Selection of HBsAg-specific DNA aptamers based on carboxylated magnetic nanoparticles and their application in the rapid and simple detection of hepatitis B virus infection. ACS Appl Mater Interfaces.

[89] Lee S, Kim YS, Jo M, Jin M, Lee DK, Kim S (2007). Chip-based detection of hepatitis C virus using RNA aptamers that specifically bind to HCV core antigen. Biochem Biophys Res Commun.

[90] Shi S, Yu X, Gao Y, Xue B, Wu X, Wang X (2014). Inhibition of hepatitis C virus production by aptamers against the core protein. J Virol.

[91] Ghanbari K, Roushani M, Azadbakht A (2017). Ultra-sensitive aptasensor based on a GQD nanocomposite for detection of hepatitis C virus core antigen. Anal Biochem.

[92] Xu M, Wang D, Wang H, Zhang X, Liang T, Dai J (2020). COVID-19 diagnostic testing: Technology perspective. Clin Transl Med.

[93] Wang C, Liu M, Wang Z, Li S, Deng Y, He N (2021). Point-of-care diagnostics for infectious diseases: From methods to devices. Nano Today.

[94] Acquah C, Jeevanandam J, Tan KX, Danquah MK (2021). Engineered aptamers for enhanced COVID-19 theranostics. Cell Mol Bioeng.

[95] Jang KJ, Lee NR, Yeo WS, Jeong YJ, Kim DE (2008). Isolation of inhibitory RNA aptamers against severe acute respiratory syndrome (SARS) coronavirus NTPase/Helicase. Biochem Biophys Res Commun.

[96] Cho SJ, Woo HM, Kim KS, Oh JW, Jeong YJ (2011). Novel system for detecting SARS coronavirus nucleocapsid protein using an ssDNA aptamer. J Biosci Bioeng.

[97] Chen Z, Wu Q, Chen J, Ni X, Dai J (2020). A DNA aptamer based Method for detection of SARS-CoV-2 nucleocapsid protein. Virol Sin.

[98] Zhang L, Fang X, Liu X, Ou H, Zhang H, Wang J (2020). Discovery of sandwich type COVID-19 nucleocapsid protein DNA aptamers. Chem Commun (Camb).

[99] Liu R, He L, Hu Y, Luo Z, Zhang J (2020). A serological aptamer-assisted proximity ligation assay for COVID-19 diagnosis and seeking neutralizing aptamers. Chem Sci.

[100] Song Y, Song J, Wei X, Huang M, Sun M, Zhu L (2020). Discovery of aptamers targeting the receptor-binding domain of the SARS-CoV-2 spike glycoprotein. Anal Chem.

[101] Chen H, Park SG, Choi N, Kwon HJ, Kang T, Lee MK (2021). Sensitive detection of SARS-CoV-2 using a SERS-based aptasensor. ACS Sens.

[102] Woo CH, Jang S, Shin G, Jung GY, Lee JW (2020). Sensitive fluorescence detection of SARS-CoV-2 RNA in clinical samples via one-pot isothermal ligation and transcription. Nat Biomed Eng.

[103] Wang Y, Zhang Y, Chen J, Wang M, Zhang T, Luo W (2021). Detection of SARS-CoV-2 and Its mutated variants via CRISPR-Cas13-based transcription amplification. Anal Chem.

[104] Bruno JG, Carrillo MP, Richarte AM, Phillips T, Andrews C, Lee JS (2012). Development, screening, and analysis of DNA aptamer libraries potentially useful for diagnosis and passive immunity of arboviruses. BMC Research Notes.

[105] Chen HL, Hsiao WH, Lee HC, Wu SC, Cheng JW (2015). Selection and characterization of DNA aptamers targeting all four serotypes of Dengue viruses. PLoS One.

[106] Basso CR, Crulhas BP, Magro M, Vianello F, Pedrosa VA (2019). A new immunoassay of hybrid nanomater conjugated to aptamers for the detection of dengue virus. Talanta.

[107] Jung JI, Han SR, Lee SW (2018). Development of RNA aptamer that inhibits methyltransferase activity of dengue virus. Biotechnol Lett.

[108] Cnossen EJ, Silva AG, Marangon K, Arruda RA, Souza EG, Santos FA (2017). Characterization of oligonucleotide aptamers targeting the 5′-UTR from dengue virus. Future Med Chem.

[109] Shubham S, Hoinka J, Banerjee S, Swanson E, Dillard JA, Lennemann NJ (2018). A 2'FY-RNA motif defines an aptamer for ebolavirus secreted protein. Sci Rep.

[110] Giamberardino A, Labib M, Hassan EM, Tetro JA, Springthorpe S, Sattar SA (2013). Ultrasensitive norovirus detection using DNA aptasensor technology. PLoS One.

[111] Weerathunge P, Ramanathan R, Torok VA, Hodgson K, Xu Y, Goodacre R (2019). Ultrasensitive colorimetric detection of murine norovirus using NanoZyme aptasensor. Anal Chem.

[112] Escudero-Abarca BI, Suh SH, Moore MD, Dwivedi HP, Jaykus LA (2014). Selection, characterization and application of nucleic acid aptamers for the capture and detection of human norovirus strains. PLoS One.

[113] Kim B, Chung KW, Lee JH (2018). Non-stop aptasensor capable of rapidly monitoring norovirus in a sample. J Pharm Biomed Anal.

[114] Chand R, Neethirajan S (2017). Microfluidic platform integrated with graphene-gold nano-composite aptasensor for one-step detection of norovirus. Biosens Bioelectron.

[115] Szakacs Z, Meszaros T, de Jonge MI, Gyurcsanyi RE (2018). Selective counting and sizing of single virus particles using fluorescent aptamer-based nanoparticle tracking analysis. Nanoscale.

[116] Yu Q, Liu M, Wu S, Xiao H, Qin X, Li P (2021). Generation and characterization of aptamers against grass carp reovirus infection for the development of rapid detection assay. J Fish Dis.

[117] Liu J, Qin Q, Zhang X, Li C, Yu Y, Huang X (2020). Development of a novel lateral flow biosensor combined with aptamer-based isolation: Application for rapid detection of grouper nervous necrosis virus. Front Microbiol.

[118] Yu Q, Liu M, Wei S, Qin X, Qin Q, Li P (2021). Research progress and prospects for the use of aptamers in aquaculture biosecurity. Aquaculture.

[119] Lautner G, Balogh Z, Bardoczy V, Meszaros T, Gyurcsanyi RE (2010). Aptamer-based biochips for label-free detection of plant virus coat proteins by SPR imaging. Analyst.

[120] Komorowska B, Hasiow-Jaroszewska B, Minicka J (2017). Application of nucleic acid aptamers for detection of Apple stem pitting virus isolates. Mol Cell Probes.

[121] Hu L, Wang L, Lu W, Zhao J, Zhang H, Chen W (2017). Selection, characterization and interaction studies of a DNA aptamer for the detection of Bifidobacterium bifidum. Int J Mol Sci.

[122] Dehghani Z, Hosseini M, Mohammadnejad J, Bakhshi B, Rezayan AH (2018). Colorimetric aptasensor for Campylobacter jejuni cells by exploiting the peroxidase like activity of Au@Pd nanoparticles. Microchim Acta.

[123] Bruno JG, Carrillo MP, Phillips T, Andrews CJ (2010). A novel screening method for competitive FRET-aptamers applied to E. coli assay development. J Fluoresc.

[124] Yu X, Chen F, Wang R, Li Y (2018). Whole-bacterium SELEX of DNA aptamers for rapid detection of E.coli O157:H7 using a QCM sensor. J Biotechnol.

[125] Suh SH, Choi SJ, Dwivedi HP, Moore MD, Escudero-Abarca BI, Jaykus LA (2018). Use of DNA aptamer for sandwich type detection of Listeria monocytogenes. Anal Biochem.

[126] Duan N, Ding X, He L, Wu S, Wei Y, Wang Z (2013). Selection, identification and application of a DNA aptamer against Listeria monocytogenes. Food Control.

[127] Ansari N, Ghazvini K, Ramezani M, Shahdordizadeh M, Yazdian-Robati R, Abnous K (2018). Selection of DNA aptamers against Mycobacterium tuberculosis Ag85A, and its application in a graphene oxide-based fluorometric assay. Microchim Acta.

[128] Dhiman A, Haldar S, Mishra SK, Sharma N, Bansal A, Ahmad Y (2018). Generation and application of DNA aptamers against HspX for accurate diagnosis of tuberculous meningitis. Tuberculosis (Edinb).

[129] Kumari P, Lavania S, Tyagi S, Dhiman A, Rath D, Anthwal D (2019). A novel aptamer-based test for the rapid and accurate diagnosis of pleural tuberculosis. Anal Biochem.

[130] Lavania S, Das R, Dhiman A, Myneedu VP, Verma A, Singh N (2018). Aptamer-based TB antigen tests for the rapid diagnosis of pulmonary tuberculosis: Potential utility in screening for tuberculosis. ACS Infect Dis.

[131] Mirzakhani K, Mousavi Gargari SL, Rasooli I, Rasoulinejad S (2017). Development of a DNA Aptamer for screening Neisseria meningitidis serogroup B by cell SELEX. Iran Biomed J.

[132] Yao W, Shi J, Ling J, Guo Y, Ding C, Ding Y (2020). SiC-functionalized fluorescent aptasensor for determination of Proteus mirabilis. Microchim Acta.

[133] Zhong Z, Gao X, Gao R, Jia L (2018). Selective capture and sensitive fluorometric determination of Pseudomonas aeruginosa by using aptamer modified magnetic nanoparticles. Microchim Acta.

[134] Pathania PK, Saini JK, Vij S, Tewari R, Sabherwal P, Rishi P (2018). Aptamer functionalized MoS2-rGO nanocomposite based biosensor for the detection of Vi antigen. Biosens Bioelectron.

[135] Wang L, Huo X, Qi W, Xia Z, Li Y, Lin J (2020). Rapid and sensitive detection of Salmonella typhimurium using nickel nanowire bridge for electrochemical impedance amplification. Talanta.

[136] Bayrac C, Eyidogan F, Avni Oktem H (2017). DNA aptamer-based colorimetric detection platform for Salmonella Enteritidis. Biosens Bioelectron.

[137] Zarei SS, Soleimanian-Zad S, Ensafi AA (2018). An impedimetric aptasensor for Shigella dysenteriae using a gold nanoparticle-modified glassy carbon electrode. Microchim Acta.

[138] Hernandez R, Valles C, Benito AM, Maser WK, Rius FX, Riu J (2014). Graphene-based potentiometric biosensor for the immediate detection of living bacteria. Biosens Bioelectron.

[139] Ranjbar S, Shahrokhian S (2018). Design and fabrication of an electrochemical aptasensor using Au nanoparticles/carbon nanoparticles/cellulose nanofibers nanocomposite for rapid and sensitive detection of Staphylococcus aureus. Bioelectrochemistry.

[140] Shin WR, Sekhon SS, Rhee SK, Ko JH, Ahn JY, Min J (2018). Aptamer-based paper strip sensor for detecting Vibrio fischeri. ACS Comb Sci.

[141] Sun Y, Duan N, Ma P, Liang Y, Zhu X, Wang Z (2019). Colorimetric aptasensor based on truncated aptamer and trivalent DNAzyme for Vibrio parahemolyticus determination. J Agric Food Chem.

[142] Wu S, Wang Y, Duan N, Ma H, Wang Z (2015). Colorimetric aptasensor based on enzyme for the detection of Vibrio parahemolyticus. J Agric Food Chem.

[143] Yan W, Gu L, Liu S, Ren W, Lyu M, Wang S (2018). Identification of a highly specific DNA aptamer for Vibrio vulnificus using systematic evolution of ligands by exponential enrichment coupled with asymmetric PCR. J Fish Dis.

[144] Bruno J, Chanpong J Methods of producing competitive aptamer fret reagents and assays. US20060257915A1, 2008

[145] Wu W, Zhang J, Zheng M, Zhong Y, Yang J, Zhao Y (2012). An aptamer-based biosensor for colorimetric detection of Escherichia coli O157:H7. PLoS One.

[146] Wang L, Huang F, Cai G, Yao L, Zhang H, Lin J (2017). An electrochemical aptasensor using coaxial capillary with magnetic nanoparticle, urease catalysis and PCB electrode for rapid and sensitive detection of Escherichia coli O157:H7. Nanotheranostics.

[147] Wu W, Zhao S, Mao Y, Fang Z, Lu X, Zeng L (2015). A sensitive lateral flow biosensor for Escherichia coli O157:H7 detection based on aptamer mediated strand displacement amplification. Anal Chim Acta.

[148] Shen H, Wang J, Liu H, Li Z, Jiang F, Wang FB (2016). Rapid and selective detection of pathogenic bacteria in bloodstream infections with aptamer-based recognition. ACS Appl Mater Interfaces.

[149] Dong X, Shi Z, Xu C, Yang C, Chen F, Lei M (2020). CdS quantum dots/Au nanoparticles/ZnO nanowire array for self-powered photoelectrochemical detection of Escherichia coli O157:H7. Biosens Bioelectron.

[150] Bruno JG, Carrillo MP, Phillips T (2008). In vitro antibacterial effects of antilipopolysaccharide DNA aptamer-C1qrs complexes. Folia Microbiol.

[151] Xie P, Zhu L, Shao X, Huang K, Tian J, Xu W (2016). Highly sensitive detection of lipopolysaccharides using an aptasensor based on hybridization chain reaction. Sci Rep.

[152] Zhu L, Li S, Shao X, Feng Y, Xie P, Luo Y (2019). Colorimetric detection and typing of E. coli lipopolysaccharides based on a dual aptamer-functionalized gold nanoparticle probe. Microchim Acta.

[153] Li H, Ding X, Peng Z, Deng L, Wang D, Chen H (2011). Aptamer selection for the detection of Escherichia coli K88. Can J Microbiol.

[154] Kim YS, Song MY, Jurng J, Kim BC (2013). Isolation and characterization of DNA aptamers against Escherichia coli using a bacterial cell-systematic evolution of ligands by exponential enrichment approach. Anal Biochem.

[155] Kim YS, Chung J, Song MY, Jurng J, Kim BC (2014). Aptamer cocktails: enhancement of sensing signals compared to single use of aptamers for detection of bacteria. Biosens Bioelectron.

[156] Jin B, Wang S, Lin M, Jin Y, Zhang S, Cui X (2017). Upconversion nanoparticles based FRET aptasensor for rapid and ultrasenstive bacteria detection. Biosens Bioelectron.

[157] Hua R, Hao N, Lu J, Qian J, Liu Q, Li H (2018). A sensitive potentiometric resolved ratiometric photoelectrochemical aptasensor for Escherichia coli detection fabricated with non-metallic nanomaterials. Biosens Bioelectron.

[158] Duan N, Wu S, Zhu C, Ma X, Wang Z, Yu Y (2012). Dual-color upconversion fluorescence and aptamer-functionalized magnetic nanoparticles-based bioassay for the simultaneous detection of Salmonella Typhimurium and Staphylococcus aureus. Anal Chim Acta.

[159] Gao W, Li B, Yao R, Li Z, Wang X, Dong X (2017). Intuitive label-free SERS detection of bacteria using aptamer-based in situ silver nanoparticles synthesis. Anal Chem.

[160] Hao L, Gu H, Duan N, Wu S, Ma X, Xia Y (2017). A chemiluminescent aptasensor based on rolling circle amplification and Co(2+)/N-(aminobutyl)-N-(ethylisoluminol) functional flowerlike gold nanoparticles for Salmonella typhimurium detection. Talanta.

[161] Ma X, Song L, Zhou N, Xia Y, Wang Z (2017). A novel aptasensor for the colorimetric detection of S. typhimurium based on gold nanoparticles. Int J Food Microbiol.

[162] Muniandy S, Dinshaw IJ, Teh SJ, Lai CW, Ibrahim F, Thong KL (2017). Graphene-based label-free electrochemical aptasensor for rapid and sensitive detection of foodborne pathogen. Anal Bioanal Chem.

[163] Bayramoglu G, Ozalp VC, Dincbal U, Arica MY (2018). Fast and sensitive detection of salmonella in milk samples using aptamer-functionalized magnetic silica solid phase and MCM-41-aptamer gate system. ACS Biomater Sci Eng.

[164] Xu X, Ma X, Wang H, Wang Z (2018). Aptamer based SERS detection of Salmonella typhimurium using DNA-assembled gold nanodimers. Microchim Acta.

[165] Muniandy S, Teh SJ, Appaturi JN, Thong KL, Lai CW, Ibrahim F (2019). A reduced graphene oxide-titanium dioxide nanocomposite based electrochemical aptasensor for rapid and sensitive detection of Salmonella enterica. Bioelectrochemistry.

[166] Ren J, Liang G, Man Y, Li A, Jin X, Liu Q (2019). Aptamer-based fluorometric determination of Salmonella Typhimurium using Fe3O4 magnetic separation and CdTe quantum dots. PLoS One.

[167] Appaturi JN, Pulingam T, Thong KL, Muniandy S, Ahmad N, Leo BF (2020). Rapid and sensitive detection of Salmonella with reduced graphene oxide-carbon nanotube based electrochemical aptasensor. Anal Biochem.

[168] Hasan MR, Pulingam T, Appaturi JN, Zifruddin AN, Teh SJ, Lim TW (2018). Carbon nanotube-based aptasensor for sensitive electrochemical detection of whole-cell Salmonella. Anal Biochem.

[169] Duan N, Wu S, Chen X, Huang Y, Xia Y, Ma X (2013). Selection and characterization of aptamers against Salmonella typhimurium using whole-bacterium systemic evolution of ligands by exponential enrichment (SELEX). J Agric Food Chem.

[170] Wang L, Wang R, Chen F, Jiang T, Wang H, Slavik M (2017). QCM-based aptamer selection and detection of Salmonella typhimurium. Food Chem.

[171] Wang L, Wang R, Wang H, Slavik M, Wei H, Li Y (2017). An aptamer-based PCR method coupled with magnetic immunoseparation for sensitive detection of Salmonella Typhimurium in ground turkey. Anal Biochem.

[172] Kolovskaya OS, Savitskaya AG, Zamay TN, Reshetneva IT, Zamay GS, Erkaev EN (2013). Development of bacteriostatic DNA aptamers for salmonella. J Med Chem.

[173] Shin WR, Sekhon SS, Kim SG, Rhee SJ, Yang GN, Won K (2018). Aptamer-based pathogen monitoring for Salmonella enterica ser. Typhimurium. J Biomed Nanotechnol.

[174] Qin L, Zheng R, Ma Z, Feng Y, Liu Z, Yang H (2009). The selection and application of ssDNA aptamers against MPT64 protein in Mycobacterium tuberculosis. Clin Chem Lab Med.

[175] Bai L, Chen Y, Bai Y, Chen Y, Zhou J, Huang A (2017). Fullerene-doped polyaniline as new redox nanoprobe and catalyst in electrochemical aptasensor for ultrasensitive detection of Mycobacterium tuberculosis MPT64 antigen in human serum. Biomaterials.

[176] Das R, Dhiman A, Mishra SK, Haldar S, Sharma N, Bansal A (2019). Structural switching electrochemical DNA aptasensor for the rapid diagnosis of tuberculous meningitis. Int J Nanomedicine.

[177] Bose JL, Bayles KW (2013). Staphylococcus aureus. In Maloy, S.; Hughes, K, Ed. Brenner's Encyclopedia of Genetics (Second Edition). San Diego, CA, USA: Academic Press.

[178] Chang YC, Yang CY, Sun RL, Cheng YF, Kao WC, Yang PC (2013). Rapid single cell detection of Staphylococcus aureus by aptamer-conjugated gold nanoparticles. Sci Rep.

[179] Cao X, Li S, Chen L, Ding H, Xu H, Huang Y (2009). Combining use of a panel of ssDNA aptamers in the detection of Staphylococcus aureus. Nucleic Acids Res.

[180] dos Santos SR, Rodrigues Correa C, Branco de Barros AL, Serakides R, Fernandes SO, Cardoso VN (2015). Identification of Staphylococcus aureus infection by aptamers directly radiolabeled with technetium-99m. Nucl Med Biol.

[181] Wu S, Duan N, Shi Z, Fang C, Wang Z (2014). Simultaneous aptasensor for multiplex pathogenic bacteria detection based on multicolor upconversion nanoparticles labels. Anal Chem.

[182] Cui F, Sun J, de Dieu Habimana J, Yang X, Ji J, Zhang Y (2019). Ultrasensitive fluorometric angling determination of Staphylococcus aureus in vitro and fluorescence imaging in vivo using carbon dots with full-color emission. Anal Chem.

[183] Moon J, Kim G, Park SB, Lim J, Mo C (2015). Comparison of whole-cell SELEX methods for the identification of Staphylococcus aureus-specific DNA aptamers. Sensors (Basel).

[184] He X, Li Y, He D, Wang K, Shangguan J, Shi H (2014). Aptamer-fluorescent silica nanoparticles bioconjugates based dual-color flow cytometry for specific detection of Staphylococcus aureus. J Biomed Nanotechnol.

[185] Chang T, Wang L, Zhao K, Ge Y, He M, Li G (2016). Duplex identification of Staphylococcus aureus by aptamer and gold nanoparticles. J Nanosci Nanotechnol.

[186] Cheng D, Yu M, Fu F, Han W, Li G, Xie J (2016). Dual recognition strategy for specific and sensitive detection of bacteria using aptamer-coated magnetic beads and antibiotic-capped gold nanoclusters. Anal Chem.

[187] Xu J, Guo J, Maina SW, Yang Y, Hu Y, Li X (2018). An aptasensor for staphylococcus aureus based on nicking enzyme amplification reaction and rolling circle amplification. Anal Biochem.

[188] Bayrac C, Oktem HA (2017). Evaluation of Staphylococcus aureus DNA aptamer by enzyme-linked aptamer assay and isothermal titration calorimetry. J Mol Recognit.

[189] Sheng L, Lu Y, Deng S, Liao X, Zhang K, Ding T (2019). A transcription aptasensor: amplified, label-free and culture-independent detection of foodborne pathogens via light-up RNA aptamers. Chem Commun (Camb).

[190] Qiao J, Meng X, Sun Y, Li Q, Zhao R, Zhang Y (2018). Aptamer-based fluorometric assay for direct identification of methicillin-resistant Staphylococcus aureus from clinical samples. J Microbiol Methods.

[191] Reich P, Stoltenburg R, Strehlitz B, Frense D, Beckmann D (2017). Development of an impedimetric aptasensor for the detection of Staphylococcus aureus. Int J Mol Sci.

[192] Stoltenburg R, Krafcikova P, Viglasky V, Strehlitz B (2016). G-quadruplex aptamer targeting protein A and its capability to detect Staphylococcus aureus demonstrated by ELONA. Sci Rep.

[193] Stoltenburg R, Schubert T, Strehlitz B (2015). In vitro selection and interaction studies of a DNA aptamer targeting protein A. PLoS One.

[194] Duan N, Wu S, Chen X, Huang Y, Wang Z (2012). Selection and identification of a DNA aptamer targeted to Vibrio parahemolyticus. J Agric Food Chem.

[195] Duan N, Wu S, Ma X, Xia Y, Wang Z (2014). A universal fluorescent aptasensor based on AccuBlue dye for the detection of pathogenic bacteria. Anal Biochem.

[196] Duan N, Wu S, Zhang H, Zou Y, Wang Z (2018). Fluorometric determination of Vibrio parahaemolyticus using an F0F1-ATPase-based aptamer and labeled chromatophores. Microchim Acta.

[197] Liu D, Hu B, Peng D, Lu S, Gao S, Li Z (2020). Isolation ssDNA aptamers specific for both live and viable but nonculturable state Vibrio vulnificus using whole bacteria-SEILEX technology. RSC Adv.

[198] Dwivedi HP, Smiley RD, Jaykus LA (2010). Selection and characterization of DNA aptamers with binding selectivity to Campylobacter jejuni using whole-cell SELEX. Appl Microbiol Biotechnol.

[199] Lamont EA, Wang P, Enomoto S, Borewicz K, Abdallah A, Isaacson RE (2014). A combined enrichment and aptamer pulldown assay for Francisella tularensis detection in food and environmental matrices. PLoS One.

[200] Bitaraf FS, Rasooli I, Mousavi Gargari SL (2016). DNA aptamers for the detection of Haemophilus influenzae type b by cell SELEX. Eur J Clin Microbiol Infect Dis.

[201] Suh SH, Dwivedi HP, Choi SJ, Jaykus LA (2014). Selection and characterization of DNA aptamers specific for Listeria species. Anal Biochem.

[202] Ohk SH, Koo OK, Sen T, Yamamoto CM, Bhunia AK (2010). Antibody-aptamer functionalized fibre-optic biosensor for specific detection of Listeria monocytogenes from food. J Appl Microbiol.

[203] Ding J, Lei J, Ma X, Gong J, Qin W (2014). Potentiometric aptasensing of Listeria monocytogenes using protamine as an indicator. Anal Chem.

[204] Park JP, Shin HJ, Park SG, Oh HK, Choi CH, Park HJ (2015). Screening and development of DNA aptamers specific to several oral pathogens. J Microbiol Biotechnol.

[205] Savory N, Lednor D, Tsukakoshi K, Abe K, Yoshida W, Ferri S (2013). In silico maturation of binding-specificity of DNA aptamers against Proteus mirabilis. Biotechnol Bioeng.

[206] Wang KY, Zeng YL, Yang XY, Li WB, Lan XP (2011). Utility of aptamer-fluorescence in situ hybridization for rapid detection of Pseudomonas aeruginosa. Eur J Clin Microbiol Infect Dis.

[207] Duan N, Ding X, Wu S, Xia Y, Ma X, Wang Z (2013). In vitro selection of a DNA aptamer targeted against Shigella dysenteriae. J Microbiol Methods.

[208] Song MS, Sekhon SS, Shin WR, Kim HC, Min J, Ahn JY (2017). Detecting and discriminating Shigella sonnei using an aptamer-based fluorescent biosensor platform. Molecules.

[209] Bruno JG, Carrillo MP (2012). Development of aptamer beacons for rapid presumptive detection of Bacillus spores. J Fluoresc.

[210] Liu Y, Jiang W, Yang S, Hu J, Lu H, Han W (2019). Rapid detection of mycoplasma-infected cells by an ssDNA aptamer probe. ACS Sens.

[211] Lee S, Kim BW, Shin HS, Go A, Lee MH, Lee DK (2019). Aptamer affinity-bead mediated capture and displacement of Gram-negative bacteria using acoustophoresis. Micromachines (Basel).

[212] Shin HS, Gedi V, Kim JK, Lee DK (2019). Detection of Gram-negative bacterial outer membrane vesicles using DNA aptamers. Sci Rep.

[213] Guo X, Wen F, Zheng N, Saive M, Fauconnier ML, Wang J (2020). Aptamer-based biosensor for detection of mycotoxins. Front Chem.

[214] Xia X, Li M, Wang M, Gu M-Q, Chi K-N, Yang Y-H (2020). Development of ochratoxin a aptasensor based on Au nanoparticles@g-C3N4. J Biomed Nanotechnol.

[215] Wang C, Sun L, Zhao Q (2019). A simple aptamer molecular beacon assay for rapid detection of aflatoxin B1. Chin Chem Lett.

[216] Tang XL, Hua Y, Guan Q, Yuan CH (2016). Improved detection of deeply invasive candidiasis with DNA aptamers specific binding to (1->3)-beta-D-glucans from Candida albicans. Eur J Clin Microbiol Infect Dis.

[217] Kinman T Parasitic Infections. https://www.healthline.com/health/parasitic-infections (12/5/2020),

[218] Ospina-Villa JD, Lopez-Camarillo C, Castanon-Sanchez CA, Soto-Sanchez J, Ramirez-Moreno E, Marchat LA (2018). Advances on aptamers against protozoan parasites. Genes (Basel).

[219] Singh NK, Chakma B, Jain P, Goswami P (2018). Protein-induced fluorescence enhancement based detection of Plasmodium falciparum glutamate dehydrogenase using carbon dot coupled specific aptamer. ACS Comb Sci.

[220] Singh NK, Jain P, Das S, Goswami P (2019). Dye coupled aptamer-captured enzyme catalyzed reaction for detection of Pan Malaria and P. falciparum species in laboratory settings and instrument-free paper-based platform. Anal Chem.

[221] Singh NK, Thungon PD, Estrela P, Goswami P (2019). Development of an aptamer-based field effect transistor biosensor for quantitative detection of Plasmodium falciparum glutamate dehydrogenase in serum samples. Biosens Bioelectron.

[222] Joseph DF, Nakamoto JA, Garcia Ruiz OA, Penaranda K, Sanchez-Castro AE, Castillo PS (2019). DNA aptamers for the recognition of HMGB1 from Plasmodium falciparum. PLoS One.

[223] Minopoli A, Della Ventura B, Lenyk B, Gentile F, Tanner JA, Offenhausser A (2020). Ultrasensitive antibody-aptamer plasmonic biosensor for malaria biomarker detection in whole blood. Nat Commun.

[224] Oteng EK, Gu W, McKeague M (2020). High-efficiency enrichment enables identification of aptamers to circulating Plasmodium falciparum-infected erythrocytes. Sci Rep.

[225] Lantero E, Belavilas-Trovas A, Biosca A, Recolons P, Moles E, Sulleiro E (2020). Development of DNA aptamers against Plasmodium falciparum blood stages using cell-systematic evolution of ligands by exponential enrichment. J Biomed Nanotechnol.

[226] Espiritu CAL, Justo CAC, Rubio MJ, Svobodova M, Bashammakh AS, Alyoubi AO (2018). Aptamer selection against a Trichomonas vaginalis adhesion protein for diagnostic applications. ACS Infect Dis.

[227] Frezza V, Pinto-Diez C, Fernandez G, Soto M, Martin ME, Garcia-Sacristan A (2020). DNA aptamers targeting Leishmania infantum H3 protein as potential diagnostic tools. Anal Chim Acta.

[228] Iqbal A, Labib M, Muharemagic D, Sattar S, Dixon BR, Berezovski MV (2015). Detection of Cryptosporidium parvum oocysts on fresh produce ssing DNA aptamers. PLoS One.

[229] Iqbal A, Liu J, Dixon B, Zargar B, Sattar SA (2019). Development and application of DNA-aptamer-coupled magnetic beads and aptasensors for the detection of Cryptosporidium parvum oocysts in drinking and recreational water resources. Can J Microbiol.

[230] Luo Y, Liu X, Jiang T, Liao P, Fu W (2013). Dual-aptamer-based biosensing of toxoplasma antibody. Anal Chem.

[231] Vargas-Montes M, Cardona N, Moncada DM, Molina DA, Zhang Y, Gomez-Marin JE (2019). Enzyme-linked aptamer assay (ELAA) for detection of toxoplasma ROP18 protein in human serum. Front Cell Infect Microbiol.

[232] Shen X, Cui W, Wang C, Cudjoe O, Zhao L, Tao Q (2020). SELEX-based direct enzyme-linked aptamer assay(DELAA) for diagnosis of Toxoplasmosis by detection of SAG1 antigen in sera of mice and humans. Research Square.

[233] Hansel TT, Kropshofer H, Singer T, Mitchell JA, George AJ (2010). The safety and side effects of monoclonal antibodies. Nat Rev Drug Discov.

[234] Zhou J, Rossi J (2017). Aptamers as targeted therapeutics: current potential and challenges. Nat Rev Drug Discov.

[235] Lakhin AV, Tarantul VZ, Gening LV (2013). Aptamers: Problems, solutions and prospects. Acta Naturae.

[236] Wang L, Lee JY, Gao L, Yin J, Duan Y, Jimenez LA (2019). A DNA aptamer for binding and inhibition of DNA methyltransferase 1. Nucleic Acids Res.

[237] Khan NH, Bui AA, Xiao Y, Sutton RB, Shaw RW, Wylie BJ (2019). A DNA aptamer reveals an allosteric site for inhibition in metallo-beta-lactamases. PLoS One.

[238] Hartmann R, Norby PL, Martensen PM, Jorgensen P, James MC, Jacobsen C (1998). Activation of 2'-5' oligoadenylate synthetase by single-stranded and double-stranded RNA aptamers. J Biol Chem.

[239] McNamara JO, Kolonias D, Pastor F, Mittler RS, Chen L, Giangrande PH (2008). Multivalent 4-1BB binding aptamers costimulate CD8+ T cells and inhibit tumor growth in mice. J Clin Invest.

[240] Ruff KM, Snyder TM, Liu DR (2010). Enhanced functional potential of nucleic acid aptamer libraries patterned to increase secondary structure. J Am Chem Soc.

[241] Khati M, Schuman M, Ibrahim J, Sattentau Q, Gordon S, James W (2003). Neutralization of infectivity of diverse R5 clinical isolates of human immunodeficiency virus type 1 by gp120-binding 2'F-RNA aptamers. J Virol.

[242] Dey AK, Khati M, Tang M, Wyatt R, Lea SM, James W (2005). An aptamer that neutralizes R5 strains of human immunodeficiency virus type 1 blocks gp120-CCR5 interaction. J Virol.

[243] Cohen C, Forzan M, Sproat B, Pantophlet R, McGowan I, Burton D (2008). An aptamer that neutralizes R5 strains of HIV-1 binds to core residues of gp120 in the CCR5 binding site. Virology.

[244] Mufhandu HT, Gray ES, Madiga MC, Tumba N, Alexandre KB, Khoza T (2012). UCLA1, a synthetic derivative of a gp120 RNA aptamer, inhibits entry of human immunodeficiency virus type 1 subtype C. J Virol.

[245] Zhou J, Satheesan S, Li H, Weinberg MS, Morris KV, Burnett JC (2015). Cell-specific RNA aptamer against human CCR5 specifically targets HIV-1 susceptible cells and inhibits HIV-1 infectivity. Chem Biol.

[246] Perrone R, Butovskaya E, Lago S, Garzino-Demo A, Pannecouque C, Palu G (2016). The G-quadruplex-forming aptamer AS1411 potently inhibits HIV-1 attachment to the host cell. Int J Antimicrob Agents.

[247] Jeon SH, Kayhan B, Ben-Yedidia T, Arnon R (2004). A DNA aptamer prevents influenza infection by blocking the receptor binding region of the viral hemagglutinin. J Biol Chem.

[248] Gopinath SC, Misono TS, Kawasaki K, Mizuno T, Imai M, Odagiri T (2006). An RNA aptamer that distinguishes between closely related human influenza viruses and inhibits haemagglutinin-mediated membrane fusion. J Gen Virol.

[249] Park SY, Kim S, Yoon H, Kim KB, Kalme SS, Oh S (2011). Selection of an antiviral RNA aptamer against hemagglutinin of the subtype H5 avian influenza virus. Nucleic Acid Ther.

[250] Kwon HM, Lee KH, Han BW, Han MR, Kim DH, Kim DE (2014). An RNA aptamer that specifically binds to the glycosylated hemagglutinin of avian influenza virus and suppresses viral infection in cells. PLoS One.

[251] Cleri F, Lensink MF, Blossey R (2020). DNA aptamers block the receptor binding domain at the spike protein of SARS-CoV-2. ChemRxiv. Preprint.

[252] Sun M, Liu S, Wei X, Wan S, Huang M, Song T (2021). Aptamer blocking strategy inhibits SARS-CoV-2 virus infection. Angew Chem Int Ed.

[253] Schmitz A, Weber A, Bayin M, Breuers S, Fieberg V, Famulok M (2021). A SARS-CoV-2 spike binding DNA aptamer that inhibits pseudovirus infection by an rbd-independent mechanism. Angew Chem Int Ed.

[254] Yang G, Li Z, Mohammed I, Zhao L, Wei W, Xiao H (2021). Identification of SARS-CoV-2-against aptamer with high neutralization activity by blocking the RBD domain of spike protein 1. Signal Transduct Target Ther.

[255] Gopinath SC, Hayashi K, Kumar PK (2012). Aptamer that binds to the gD protein of herpes simplex virus 1 and efficiently inhibits viral entry. J Virol.

[256] Yadavalli T, Agelidis A, Jaishankar D, Mangano K, Thakkar N, Penmetcha K (2017). Targeting herpes simplex virus-1 gD by a DNA aptamer can be an effective new strategy to curb viral infection. Mol Ther Nucleic Acids.

[257] Yang D, Meng X, Yu Q, Xu L, Long Y, Liu B (2013). Inhibition of hepatitis C virus infection by DNA aptamer against envelope protein. Antimicrob Agents Chemother.

[258] Gandham SH, Volk DE, Lokesh GL, Neerathilingam M, Gorenstein DG (2014). Thioaptamers targeting dengue virus type-2 envelope protein domain III. Biochem Biophys Res Commun.

[259] Xu J, Zhang X, Zhou S, Shen J, Yang D, Wu J (2017). A DNA aptamer efficiently inhibits the infectivity of Bovine herpesvirus 1 by blocking viral entry. Sci Rep.

[260] Liang H, Fu X, Liu L, Lin Q, Guo H, Li Y (2017). Inhibition of grass carp reovirus infection by DNA aptamers against S10 protein. J Aquat Anim Health.

[261] Kalra P, Mishra SK, Kaur S, Kumar A, Prasad HK, Sharma TK (2018). G-quadruplex-forming DNA aptamers inhibit the DNA-binding function of HupB and Mycobacterium tuberculosis entry into host cells. Mol Ther Nucleic Acids.

[262] Rando RF, Ojwang J, Elbaggari A, Reyes GR, Tinder R, McGrath MS (1995). Suppression of human immunodeficiency virus type 1 activity in vitro by oligonucleotides which form intramolecular tetrads. J Biol Chem.

[263] Ojwang JO, Buckheit RW, Pommier Y, Mazumder A, Vreese KD, J A Esté DR (1995). T30177, an oligonucleotide stabilized by an intramolecular guanosine octet, is a potent inhibitor of laboratory strains and clinical isolates of human immunodeficiency virus type 1. Antimicrob Agents Chemother.

[264] Virgilio A, Amato T, Petraccone L, Esposito F, Grandi N, Tramontano E (2018). Improvement of the activity of the anti-HIV-1 integrase aptamer T30175 by introducing a modified thymidine into the loops. Sci Rep.

[265] Schneider DJ, Feigon J, Hostomsky Z, Gold L (1995). High-affinity ssDNA inhibitors of the reverse transcriptase of type 1 human immunodeficiency virus. Biochemistry.

[266] Symensma TL, Giver L, Zapp M, Takle GB, Ellington AD (1996). RNA aptamers selected to bind human immunodeficiency virus type 1 Rev in vitro are Rev responsive in vivo. J Virol.

[267] Michalowski D, Chitima-Matsiga R, Held DM, Burke DH (2008). Novel bimodular DNA aptamers with guanosine quadruplexes inhibit phylogenetically diverse HIV-1 reverse transcriptases. Nucleic Acids Res.

[268] Li N, Wang Y, Pothukuchy A, Syrett A, Husain N, Gopalakrisha S (2008). Aptamers that recognize drug-resistant HIV-1 reverse transcriptase. Nucleic Acids Res.

[269] Nguyen PDM, Zheng J, Gremminger TJ, Qiu L, Zhang D, Tuske S (2020). Binding interface and impact on protease cleavage for an RNA aptamer to HIV-1 reverse transcriptase. Nucleic Acids Res.

[270] Ramalingam D, Duclair S, Datta SA, Ellington A, Rein A, Prasad VR (2011). RNA aptamers directed to human immunodeficiency virus type 1 Gag polyprotein bind to the matrix and nucleocapsid domains and inhibit virus production. J Virol.

[271] Duclair S, Gautam A, Ellington A, Prasad VR (2015). High-affinity RNA aptamers against the HIV-1 protease inhibit both in vitro protease activity and late events of viral replication. Mol Ther Nucleic Acids.

[272] Lee CH, Lee YJ, Kim JH, Lim JH, Kim JH, Han W (2013). Inhibition of hepatitis C virus (HCV) replication by specific RNA aptamers against HCV NS5B RNA replicase. J Virol.

[273] Gao Y, Yu X, Xue B, Zhou F, Wang X, Yang D (2014). Inhibition of hepatitis C virus infection by DNA aptamer against NS2 protein. PLoS One.

[274] Yu X, Gao Y, Xue B, Wang X, Yang D, Qin Y (2014). Inhibition of hepatitis C virus infection by NS5A-specific aptamer. Antiviral Res.

[275] Orabi A, Bieringer M, Geerlof A, Bruss V (2015). An aptamer against the matrix binding domain on the hepatitis B virus capsid impairs virion formation. J Virol.

[276] Han SR, Lee SW (2017). Inhibition of Japanese encephalitis virus (JEV) replication by specific RNA aptamer against JEV methyltransferase. Biochem Biophys Res Commun.

[277] Zhou L, Li P, Yang M, Yu Y, Huang Y, Wei J (2016). Generation and characterization of novel DNA aptamers against coat protein of grouper nervous necrosis virus (GNNV) with antiviral activities and delivery potential in grouper cells. Antiviral Res.

[278] Valencia-Resendiz DG, Palomino-Vizcaino G, Tapia-Vieyra JV, Benıtez-Hess ML, Leija-Montoya AG, Alvarez-Salas LM (2018). Inhibition of human papillomavirus type 16 infection using an RNA aptamer. Nucleic Acid Ther.

[279] Xu J, Cai Y, Jiang B, Li X, Jin H, Liu W (2019). An optimized aptamer-binding viral tegument protein VP8 inhibits the production of Bovine Herpesvirus-1 through blocking nucleocytoplasmic shuttling. Int J Biol Macromol.

[280] Leija-Montoya AG, Benitez-Hess ML, Toscano-Garibay JD, Alvarez-Salas LM (2014). Characterization of an RNA aptamer against HPV-16 L1 virus-like particles. Nucleic Acid Ther.

[281] Shum KT, Lui EL, Wong SC, Yeung P, Sam L, Wang Y (2011). Aptamer-mediated inhibition of Mycobacterium tuberculosis polyphosphate kinase 2. Biochemistry.

[282] Bachtiar BM, Srisawat C, Bachtiar EW (2019). RNA aptamers selected against yeast cells inhibit Candida albicans biofilm formation in vitro. Microbiologyopen.

[283] Vahed M, Ahmadian G, Ameri N, Vahed M (2019). G-rich VEGF aptamer as a potential inhibitor of chitin trafficking signal in emerging opportunistic yeast infection. Comput Biol Chem.

[284] Ospina-Villa JD, Dufour A, Weber C, Ramirez-Moreno E, Zamorano-Carrillo A, Guillen N (2018). Targeting the polyadenylation factor EhCFIm25 with RNA aptamers controls survival in Entamoeba histolytica. Sci Rep.

[285] Chang TW, Blank M, Janardhanan P, Singh BR, Mello C, Blind M (2010). In vitro selection of RNA aptamers that inhibit the activity of type A botulinum neurotoxin. Biochem Biophys Res Commun.

[286] Vivekananda J, Salgado C, Millenbaugh NJ (2014). DNA aptamers as a novel approach to neutralize Staphylococcus aureus alpha-toxin. Biochem Biophys Res Commun.

[287] Wang K, Wu D, Chen Z, Zhang X, Yang X, Yang CJ (2016). Inhibition of the superantigenic activities of Staphylococcal enterotoxin A by an aptamer antagonist. Toxicon.

[288] Wang K, Gan L, Jiang L, Zhang X, Yang X, Chen M (2015). Neutralization of staphylococcal enterotoxin B by an aptamer antagonist. Antimicrob Agents Chemother.

[289] El-Aziz TMA, Ravelet C, Molgo J, Fiore E, Pale S, Amar M (2017). Efficient functional neutralization of lethal peptide toxins in vivo by oligonucleotides. Sci Rep.

[290] Lahousse M, Park HC, Lee SC, Ha NR, Jung IP, Schlesinger SR (2018). Inhibition of anthrax lethal factor by ssDNA aptamers. Arch Biochem Biophys.

[291] Esposito CL, Catuogno S, Condorelli G, Ungaro P, de Franciscis V (2018). Aptamer chimeras for therapeutic delivery: The challenging perspectives. Genes (Basel).

[292] Bobbin ML, Burnett JC, Rossi JJ (2015). RNA interference approaches for treatment of HIV-1 infection. Genome Med.

[293] Zhou J, Li H, Li S, Zaia J, Rossi JJ (2008). Novel dual inhibitory function aptamer-siRNA delivery system for HIV-1 therapy. Mol Ther.

[294] Zhou J, Swiderski P, Li H, Zhang J, Neff CP, Akkina R (2009). Selection, characterization and application of new RNA HIV gp 120 aptamers for facile delivery of Dicer substrate siRNAs into HIV infected cells. Nucleic Acids Res.

[295] Neff CP, Zhou J, Remling L, Kuruvilla J, Zhang J, Li H (2011). An aptamer-siRNA chimera suppresses HIV-1 viral loads and protects from helper CD4(+) T cell decline in humanized mice. Sci Transl Med.

[296] Zhou J, Neff CP, Swiderski P, Li H, Smith DD, Aboellail T (2013). Functional in vivo delivery of multiplexed anti-HIV-1 siRNAs via a chemically synthesized aptamer with a sticky bridge. Mol Ther.

[297] Zhou J, Lazar D, Li H, Xia X, Satheesan S, Charlins P (2018). Receptor-targeted aptamer-siRNA conjugate-directed transcriptional regulation of HIV-1. Theranostics.

[298] Wheeler LA, Trifonova R, Vrbanac V, Basar E, McKernan S, Xu Z (2011). Inhibition of HIV transmission in human cervicovaginal explants and humanized mice using CD4 aptamer-siRNA chimeras. J Clin Invest.

[299] Zhou L, Wang S, Yu Q, Wei S, Liu M, Wei J (2020). Characterization of novel aptamers specifically directed to red-spotted grouper nervous necrosis virus (RGNNV)-infected cells for mediating targeted siRNA delivery. Front Microbiol.

[300] Kavruk M, Celikbicak O, Ozalp VC, Borsa BA, Hernandez FJ, Bayramoglu G (2015). Antibiotic loaded nanocapsules functionalized with aptamer gates for targeted destruction of pathogens. Chem Commun (Camb).

[301] Ucak S, Sudagidan M, Borsa BA, Mansuroglu B, Ozalp VC (2020). Inhibitory effects of aptamer targeted teicoplanin encapsulated PLGA nanoparticles for Staphylococcus aureus strains. World J Microbiol Biotechnol.

[302] Song MY, Jurng J, Park YK, Kim BC (2016). An aptamer cocktail-functionalized photocatalyst with enhanced antibacterial efficiency towards target bacteria. J Hazard Mater.

[303] Lee B, Park J, Ryu M, Kim S, Joo M, Yeom JH (2017). Antimicrobial peptide-loaded gold nanoparticle-DNA aptamer conjugates as highly effective antibacterial therapeutics against Vibrio vulnificus. Sci Rep.

[304] Soundy J, Day D (2020). Delivery of antibacterial silver nanoclusters to Pseudomonas aeruginosa using species-specific DNA aptamers. J Med Microbiol.

[305] Homann M, Goringer HU (2001). Uptake and intracellular transport of RNA Aptamers in African trypanosomes suggest therapeutic “Piggy-Back” approach. Bioorg Med Chem.

[306] Cardenas-Guerra RE, Moreno-Gutierrez DS, Vargas-Dorantes OJ, Espinoza B, Hernandez-Garcia A (2020). Delivery of antisense DNA into pathogenic parasite Trypanosoma cruzi using virus-like protein-based nanoparticles. Nucleic Acid Ther.

[307] Finlay BB, McFadden G (2006). Anti-immunology: evasion of the host immune system by bacterial and viral pathogens. Cell.

[308] Woo HM, Kim KS, Lee JM, Shim HS, Cho SJ, Lee WK (2013). Single-stranded DNA aptamer that specifically binds to the influenza virus NS1 protein suppresses interferon antagonism. Antiviral Res.

[309] Woo HM, Lee JM, Kim CJ, Lee JS, Jeong YJ (2019). Recovery of TRIM25-mediated RIG-I ubiquitination through suppression of NS1 by RNA aptamers. Mol Cells.

[310] Pan Q, Wang Q, Sun X, Xia X, Wu S, Luo F (2014). Aptamer against mannose-capped lipoarabinomannan inhibits virulent Mycobacterium tuberculosis infection in mice and rhesus monkeys. Mol Ther.

[311] Sun X, Pan Q, Yuan C, Wang Q, Tang XL, Ding K (2016). A single ssDNA aptamer binding to mannose-capped lipoarabinomannan of bacillus calmette-guerin enhances immunoprotective effect against tuberculosis. J Am Chem Soc.

[312] Tanaka K, Kasahara Y, Miyamoto Y, Okuda T, Kasai T, Onodera K (2018). Development of oligonucleotide-based antagonists of Ebola virus protein 24 inhibiting its interaction with karyopherin alpha 1. Org Biomol Chem.

[313] Wu CC, Sabet M, Hayashi T, Tawatao R, Fierer J, Carson DA (2008). In vivo efficacy of a phosphodiester TLR-9 aptamer and its beneficial effect in a pulmonary anthrax infection model. Cell Immunol.

[314] Leonard F, Ha NP, Sule P, Alexander JF, Volk DE, Lokesh GLR (2017). Thioaptamer targeted discoidal microparticles increase self immunity and reduce Mycobacterium tuberculosis burden in mice. J Control Release.

[315] Hwang SY, Sun HY, Lee KH, Oh BH, Cha YJ, Kim BH (2012). 5'-Triphosphate-RNA-independent activation of RIG-I via RNA aptamer with enhanced antiviral activity. Nucleic Acids Res.

[316] Avci-Adali M, Steinle H, Michel T, Schlensak C, Wendel HP (2013). Potential capacity of aptamers to trigger immune activation in human blood. PLoS One.

[317] Gefen T, Castro I, Muharemagic D, Puplampu-Dove Y, Patel S, Gilboa E (2017). A TIM-3 oligonucleotide aptamer enhances T cell functions and potentiates tumor immunity in mice. Mol Ther.

[318] Boshtam M, Asgary S, Kouhpayeh S, Shariati L, Khanahmad H (2017). Aptamers against pro- and anti-inflammatory cytokines: A review. Inflammation.

[319] Hu PP (2017). Recent advances in aptamers targeting immune system. Inflammation.

[320] Vorobyeva M, Timoshenko V, Vorobjev P, Venyaminova A (2016). Aptamers against immunologic targets: Diagnostic and therapeutic prospects. Nucleic Acid Ther.

[321] Adachi T, Nakamura Y (2019). Aptamers: A review of their chemical properties and modifications for therapeutic application. Molecules.

[322] Kalra P, Dhiman A, Cho WC, Bruno JG, Sharma TK (2018). Simple methods and rational design for enhancing aptamer sensitivity and specificity. Front Mol Biosci.

